# Efficacy and Safety of Anakinra Plus Standard of Care for Patients With Severe COVID-19: A Randomized Phase 2/3 Clinical Trial

**DOI:** 10.1001/jamanetworkopen.2023.7243

**Published:** 2023-04-07

**Authors:** Patricia Fanlo, Borja del Carmelo Gracia-Tello, Eva Fonseca Aizpuru, Jorge Álvarez-Troncoso, Andrés Gonzalez, Sergio Prieto-González, Mayka Freire, Ana Belén Argibay, Lucio Pallarés, José Antonio Todolí, Mercedes Pérez, Segundo Buján-Rivas, Berta Ibáñez

**Affiliations:** 1 Unidad de Enfermedades Autoinmunes Sistémicas, Servicio de Medicina Interna, Hospital Universitario de Navarra, Pamplona, Spain; 2 Unidad de Enfermedades Autoinmunes Sistémicas, Servicio de Medicina Interna, Hospital Universitario Clínico Lozano Blesa, Zaragoza, Spain; 3 Unidad de Enfermedades Autoinmunes Sistémicas, Servicio de Medicina Interna, Hospital de Cabueñes, Gijón, Spain; 4 Unidad de Enfermedades Autoinmunes Sistémicas, Servicio de Medicina Interna, Hospital La Paz, Madrid, Spain; 5 Unidad de Enfermedades Autoinmunes Sistémicas, Servicio de Medicina Interna, Hospital Ramón y Cajal, Madrid, Spain; 6 Servicio de Enfermedades Autoinmunes Sistémicas, Hospital Clinic, Barcelona, Spain; 7 Unidad de Enfermedades Autoinmunes Sistémicas, Servicio de Medicina Interna, Complejo Hospitalario de Santiago, Santiago de Compostela, Spain; 8 Unidad de Enfermedades Autoinmunes Sistémicas, Servicio de Medicina Interna, Complejo Hospitalario de Vigo, Vigo, Spain; 9 Unidad de Enfermedades Autoinmunes Sistémicas, Servicio de Medicina Interna, Hospital Son Espases, Palma de Mallorca, Spain; 10 Unidad de Enfermedades Autoinmunes Sistémicas, Servicio de Medicina Interna, Hospital Universitario La Fe, Valencia, Spain; 11 Unidad de Enfermedades Autoinmunes Sistémicas, Servicio de Medicina Interna, Hospital Universitario Miguel Servet, Zaragoza, Spain; 12 Unidad de Enfermedades Autoinmunes Sistémicas, Servicio de Medicina Interna, Hospital Vall d´Hebron, Barcelona, Spain; 13 Navarrabiomed, Hospital Universitario de Navarra, Universidad Pública de Navarra, Red de Investigación en Cronicidad, Atención Primaria y Prevención y Promoción de la Salud, Pamplona, Spain

## Abstract

**Importance:**

COVID-19 pneumonia is often associated with hyperinflammation. The efficacy and safety of anakinra in treating patients with severe COVID-19 pneumonia and hyperinflammation are still unclear.

**Objective:**

To assess the efficacy and safety of anakinra vs standard of care alone for patients with severe COVID-19 pneumonia and hyperinflammation.

**Design, Setting, and Participants:**

The Clinical Trial of the Use of Anakinra in Cytokine Storm Syndrome Secondary to COVID-19 (ANA-COVID-GEAS) was a multicenter, randomized, open-label, 2-group, phase 2/3 clinical trial conducted at 12 hospitals in Spain between May 8, 2020, and March 1, 2021, with a follow-up of 1 month. Participants were adult patients with severe COVID-19 pneumonia and hyperinflammation. Hyperinflammation was defined as interleukin-6 greater than 40 pg/mL, ferritin greater than 500 ng/mL, C-reactive protein greater than 3 mg/dL (rationale, ≥5 upper normal limit), and/or lactate dehydrogenase greater than 300 U/L. Severe pneumonia was considered if at least 1 of the following conditions was met: ambient air oxygen saturation 94% or less measured with a pulse oximeter, ratio of partial pressure O_2_ to fraction of inspired O_2_ of 300 or less, and/or a ratio of O_2_ saturation measured with pulse oximeter to fraction of inspired O_2_ of 350 or less. Data analysis was performed from April to October 2021.

**Interventions:**

Usual standard of care plus anakinra (anakinra group) or usual standard of care alone (SoC group). Anakinra was given at a dose of 100 mg 4 times a day intravenously.

**Main Outcomes and Measures:**

The primary outcome was the proportion of patients not requiring mechanical ventilation up to 15 days after treatment initiation, assessed on an intention-to-treat basis.

**Results:**

A total of 179 patients (123 men [69.9%]; mean [SD] age, 60.5 [11.5] years) were randomly assigned to the anakinra group (92 patients) or to the SoC group (87 patients). The proportion of patients not requiring mechanical ventilation up to day 15 was not significantly different between groups (64 of 83 patients [77.1%] in the anakinra group vs 67 of 78 patients [85.9%] in the SoC group; risk ratio [RR], 0.90; 95% CI, 0.77-1.04; *P* = .16). Anakinra did not result in any difference in time to mechanical ventilation (hazard ratio, 1.72; 95% CI, 0.82-3.62; *P* = .14). There was no significant difference between groups in the proportion of patients not requiring invasive mechanical ventilation up to day 15 (RR, 0.99; 95% CI, 0.88-1.11; *P* > .99).

**Conclusions and Relevance:**

In this randomized clinical trial, anakinra did not prevent the need for mechanical ventilation or reduce mortality risk compared with standard of care alone among hospitalized patients with severe COVID-19 pneumonia.

**Trial Registration:**

ClinicalTrials.gov Identifier: NCT04443881

## Key Points

**Question:**

Is the use of anakinra plus the standard of care effective compared with the standard of care alone in reducing the probability of requiring mechanical ventilation among patients with severe COVID-19 pneumonia?

**Findings:**

In this randomized clinical trial with 179 patients, the proportion of patients not requiring mechanical ventilation up to day 15 was not significantly different between groups (77.1% for anakinra group vs 85.9% for standard of care group).

**Meaning:**

This study did not find evidence of effectiveness of anakinra vs the standard of care in reducing the need for mechanical ventilation among patients with severe COVID-19 pneumonia, and future research should assess anakinra in patients with less-severe pneumonia.

## Teaser

This randomized clinical trial assesses the efficacy and safety of anakinra vs standard of care alone in patients with severe COVID-19 pneumonia and hyperinflammation.

## Introduction

COVID-19 has resulted in millions of cases worldwide. Multiple variants of SARS-CoV-2 have emerged that vary in terms of transmissibility and severity. The risk of severe outcomes following SARS-CoV-2 infection is substantially lower for the Omicron variant than for the Delta variant. Although more than 80% of patients experience mild symptoms, a substantial proportion can be critically ill, especially those who are older and those with comorbidities.^1,2,3^ The pathogenesis of COVID-19 involves a dysregulated host immune response and excessive inflammation, which can lead to acute respiratory distress syndrome and multiorgan failure.^4,5,6^ A subgroup of patients with severe COVID-19 show hyperinflammatory features, with increased circulating levels of cytokines, including interleukin (IL)–1 and IL-6,^3,5^ which are associated with poor outcomes.^7,8^

Anakinra is a recombinant IL-1 receptor antagonist that has traditionally been used in scenarios with features similar to those of COVID-19 hyperinflammation, including macrophage activation syndrome caused by severe inflammatory disorders and septic shock.^9,10,11^ The benefit of anakinra in patients with COVID-19 was evaluated in several retrospective and nonrandomized prospective studies^12,13,14,15,16,17,18,19,20^ that showed improved outcomes in terms of mortality and mechanical ventilation. Evidence from randomized clinical trials is limited.^21,22,23^ On the basis of the SAVE-MORE (suPAR-Guided Anakinra Treatment for Validation of the Risk and Early Management of Severe Respiratory Failure by COVID-19)^22^ study findings, the European Medicines Agency authorized the use of anakinra for treating adult patients with COVID-19 pneumonia requiring supplemental oxygen who are at risk of progressing to severe respiratory failure as determined by elevated soluble urokinase plasminogen activator receptor (suPAR) levels (≥6 ng/mL). The present randomized clinical trial was designed to assess the efficacy and safety of anakinra vs standard of care (SoC) alone in patients with severe COVID-19 pneumonia and hyperinflammation.

## Methods

### Trial Design and Oversight

This study was performed in accordance with the Declaration of Helsinki,^24^ the International Council for Harmonization Good Clinical Practice guidelines, and Spanish regulation. The study protocol was approved by the Ethics Committee of Clinical Research of Navarre and can be found in Supplement 1. Written informed consent was obtained from all patients before enrollment. This study follows the Consolidated Standards of Reporting Trials (CONSORT) reporting guideline for randomized clinical trials (Supplement 1).

The Clinical Trial of the Use of Anakinra in Cytokine Storm Syndrome Secondary to COVID-19 (ANA-COVID-GEAS) study was a multicenter, randomized, open-label, 2-group, phase 2/3 clinical trial designed to assess the clinical outcomes and safety of anakinra plus SoC (anakinra group) vs SoC alone (SoC group) in patients with severe COVID-19 pneumonia. The trial was decentralized and conducted at 12 hospitals in Spain (see the list of participating centers in eAppendix 1 in Supplement 2). Eligible patients were adults (aged ≥18 years) with confirmed severe COVID-19 pneumonia, defined as a positive polymerase chain reaction test for SARS-CoV-2 in nasopharyngeal specimens, pulmonary infiltrates compatible with pneumonia on chest imaging, and hyperinflammation. Hyperinflammation was defined as IL-6 greater than 40 pg/mL, ferritin greater than 500 ng/mL (to convert to micrograms per liter, multiply by 1), C-reactive protein (CRP) greater than 3.0 mg/dL (to convert to milligrams per liter, multiply by 10) (rationale, ≥5 upper normal limit), and/or lactate dehydrogenase greater than 300 U/L (to convert to microkatals per liter, multiply by 0.0167). Severe pneumonia was considered if at least 1 of the following conditions was met: ambient air oxygen saturation 94% or less measured with a pulse oximeter, ratio of partial pressure O_2_ (Po_2_) to fraction of inspired O_2_ (FiO_2_) of 300 or less, and/or ratio of O_2_ saturation measured with pulse oximeter to FiO_2_ of 350 or less. The full inclusion and exclusion criteria are detailed in eAppendix 2 of Supplement 2.

Participants were randomly assigned to receive either SoC plus anakinra (anakinra group) or SoC alone (SoC group) in 1:1 proportion. A computer-generated randomization list with permuted blocks of sizes of 10 and 12 was generated by statisticians of the Methodology Unit of Navarrabiomed using the randomizeR library of the R statistical package version 3.4.0 (R Project for Statistical Computing). Allocation sequencing was stratified and concealed by center to guarantee balance between groups within center. A web-based electronic case report form was used for the assignment. The trial participants and investigators and the statisticians who conducted the analysis were not masked to treatment assignment.

Anakinra was given intravenously (100 mg 4 times a day) for a maximum of 15 days according to clinical response based on the following criteria: the need for hospital beds given the hospital pressure during the first waves of the pandemic, the persistence of elevated inflammatory markers, the persistence of pneumonic condensation on radiologic images, or the fact that some patients continued to require oxygen therapy and were transferred home under hospitalization at home protocols. The SoC included hydroxychloroquine, lopinavir-ritonavir, and/or azithromycin. Intravenous methylprednisolone pulse therapy was permitted at any time during the study.^25,26^ Details on anakinra dose selection, SoC treatment, concomitant therapy, and rescue medication are given in eAppendix 2 of Supplement 2.

Patients were monitored during the hospital stay and then were followed up for 28 days after randomization or until premature withdrawal from the study, loss to follow-up, or death, whichever occurred first. Assessments at randomization (day 1) included vital signs, lung function, and laboratory test parameters. After treatment allocation, clinical and laboratory test parameters were assessed in the hospital on days 4, 7, and 15 and by telephone at day 28 if discharged. Details of the trial conduct and procedures can be found in eAppendix 2 of Supplement 2.

### Outcome Measures

The primary efficacy outcome was the proportion of patients not requiring mechanical ventilation (invasive or noninvasive mechanical ventilation) at 15 days after treatment initiation. Secondary outcomes were time to mechanical ventilation, change in resting oxygen saturation and oxygen supplementation from baseline to day 15, evolution of Po_2_/FiO_2_ from baseline to day 15, risk of intensive care unit (ICU) admission, and length of stay in hospital and in ICU. Additional secondary outcomes were 28-day mortality, 48-hour and 7-day in-hospital and ICU mortality, and viral clearance (negative SARS-CoV-2 RNA in nasopharyngeal swabs) by day 15. Safety outcomes included the incidence of treatment-emergent serious adverse events, adverse events leading to premature discontinuation of study treatment, and anaphylactic or anaphylactoid reactions and were coded using the Medical Dictionary for Regulatory Activities. Exploratory outcomes were evolution of levels of inflammatory markers and other relevant laboratory test parameters, including IL-6, ferritin, D-dimer, CRP, lactate dehydrogenase, and lymphocytes, from baseline to days 4, 7, and 15, and time to defervescence. Outcomes analyzed post hoc were time to mechanical ventilation or death as composite end point and proportion of patients not requiring invasive mechanical ventilation at 28 days.

### Statistical Analysis

Data analysis was performed from April to October 2021. Assuming a confidence level of 0.95, a 2-sided test, and a proportion of 78% of patients not requiring mechanical ventilation in the SoC group, an effective sample size of 90 patients per group (180 patients in total) was required to provide 0.75 power in detecting as significantly different a proportion of 92% of patients not requiring it in the anakinra group. The assumed proportion in the SoC group (78%) was based on an estimation using available data from participating hospitals, and the target difference (14%) was chosen trying to have a trade-off between clinical relevance and feasibility. Analyses of the primary and secondary outcomes and the safety analysis were performed on an intention-to-treat (ITT) basis. Exploratory outcomes were assessed for all patients who had available follow-up data at each time point. No data imputation was done to account for missing data, which accounted for only 5% of the data.

The treatment effect size for the primary and secondary end points was measured on the basis of the relative risk ratio (RR). The treatment effect was also expressed using the hazard ratio (HR) with the 95% CI for time to mechanical ventilation and mortality, plotted with Kaplan-Meier survival curves and compared with the log-rank test. Post hoc adjusted Cox regression analyses were performed and included COVID-19 severity (World Health Organization clinical progression scale score >5 vs ≤5) and use of dexamethasone as an important prognostic factor based on the RECOVERY study.^27^ These variables were also used to provide adjusted estimates for treatment effect on viral clearance in logistic regression models. We further performed a post hoc sensitivity analysis on patients with a Severe COVID Prediction Estimate (SCOPE) score 6 or higher.^28^ Mixed models for repeated measurements analyses were fitted to assess the evolution of hyperinflammatory parameters over time, using time and group as fixed effects, time-by-group as a possible interaction effect (removed if not significant), and patient as random effect. Two-sided *P* values are provided for all tests, with *P* < .05 considered significant. The analyses were performed using SPSS statistical software version 22.0 (SPSS-IBM) and R statistical software version 3.6.3 (R Project for Statistical Computing).

## Results

### Patients

**Figure 1.**
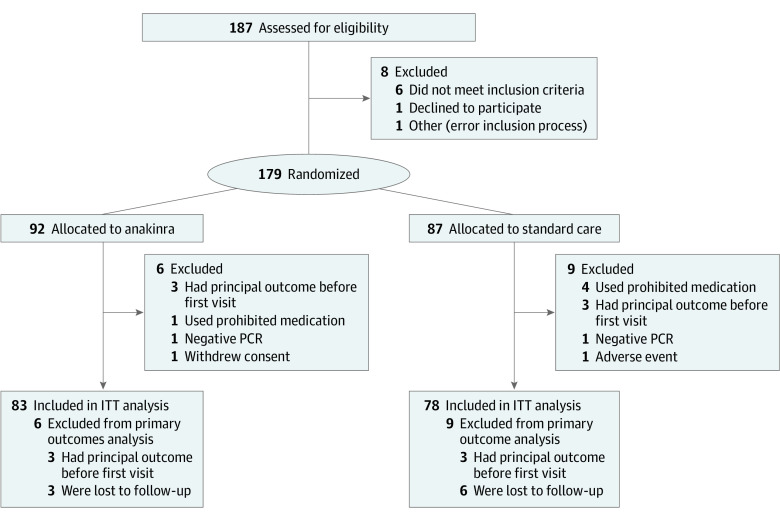
Trial Enrollment Flowchart ITT indicates intention to treat; PCR, polymerase chain reaction.

Between May 8, 2020, and March 1, 2021, a total of 187 patients were screened. Of these, 179 were randomly assigned to anakinra plus SoC (92 patients) or SoC alone (87 patients) (Figure 1). The mean time from the date of diagnosis of SARS-CoV-2 infection to the date of randomization was 6 days, and the mean time from the date of diagnosis of interstitial pneumonia to date of randomization was 2 days. Three patients assigned to anakinra withdrew consent after randomization before receiving the first dose of anakinra, and 9 patients were lost to follow-up for the primary end point; thus, 83 patients in the anakinra group and 78 patients in the SoC group were included in the ITT population for the main analyses. The patient baseline characteristics are shown in Table 1 and eTable 1 in Supplement 2. Baseline demographic and clinical characteristics were generally well-balanced between treatment groups. The mean (SD) age of the patients was 60.5 (11.5) years, and 123 (69.9%) were men. There was a higher proportion of patients receiving oxygen supplementation in the anakinra group (88 of 89 patients [98.9%]) than in the SoC group (77 of 91 patients [88.5%]), and 9 patients (5.1%; 6 in the anakinra group and 3 in the SoC group) required noninvasive ventilation. Seventy-four patients (83.1%) who received anakinra and 67 patients (77.0%) in the SoC group had a World Health Organization clinical progression scale score of 5 or less; 119 patients (66.4%) presented with a SCOPE score of 6 or higher. In addition, 36 patients (41.4%) in the SoC group experienced arterial hypertension.

**Table 1.** Baseline Demographic, Clinical, and Laboratory Characteristics of the Intention-to-Treat Population

Characteristic	Patients, No. (%)^a^		
	All patients (N = 176)	Anakinra group (n = 89)	SoC group (n = 87)
Age, mean (SD), y	60.5 (11.5)	61.1 (11.7)	59.8 (11.3)
Sex			
Male	123 (69.9)	66 (74.2)	57 (65.5)
Female	53 (30.1)	23 (25.8)	30 (34.5)
Body mass index^b^			
No.	142	73	69
Median (IQR)	28.9 (25.4-32.6)	29.4 (25.8-32.6)	28.4 (24.9-32.7)
Vital signs and respiratory status			
Temperature, °C			
No.	176	89	87
Median (IQR)	36.2 (35.8-36.5)	36.2 (36.0-36.7)	36.2 (35.7-36.4)
Respiratory rate, breaths/min			
No.	152	76	76
Median (IQR)	20.0 (17.3-24.0)	20.0 (18.0-24.0)	20.0 (16.3-23.5)
Oxygen saturation, %			
No.	75	37	38
Median (IQR)	93.0 (90.0-94.0)	93.0 (90.0-93.5)	93.0 (90.0-94.0)
Oxygen saturation <90% or respiratory rate >30 breaths/min, No. of patients/total No. (%)	16/72 (22.2)	8/35 (22.9)	8/37 (21.6)
Ratio of arterial partial pressure of oxygen to fraction of inspired oxygen			
No.	111	56	55
Median (IQR)	278.0 (216.7-318.6)	276.0 (209.5-318.9)	278.6 (223.8-306.0)
Modified Early Warning Score			
No.	174	88	86
Median (IQR)	1.0 (1.0-2.0)	1.5 (1.0-2.0)	1.0 (1.0-2.0)
Respiratory support			
Oxygen supplementation^c^	165 (93.8)	88 (98.9)	77 (88.5)
Duration of oxygen supplementation, d			
No.	165	88	77
Median (IQR)	4.0 (4.0-5.0)	4.0 (4.0-5.0)	4.0 (4.0-5.0)
Noninvasive mechanical ventilation	9 (5.1)	6 (6.7)	3 (3.4)
Time from SARS-CoV-2 infection diagnosis to randomization, d			
No.	176	89	87
Median (IQR)	6.0 (2.0-9.0)	6.0 (2.0-8.0)	6.0 (2.0-9.0)
Time from diagnosis of interstitial pneumonia on imaging to randomization, d			
No.	176	89	87
Median (IQR)	1.0 (1.0-2.8)	2.0 (1.0-3.0)	1.0 (1.0-2.0)
Comorbidities^d^			
Arterial hypertension	70 (39.8)	34 (38.2)	36 (41.4)
Chronic heart failure	30 (17.0)	13 (14.6)	17 (19.5)
Diabetes	25 (14.2)	10 (11.2)	15 (17.2)
Asthma	18 (10.2)	9 (10.1)	9 (10.3)
Chronic pulmonary disease	14 (8.0)	6 (6.7)	8 (9.2)
Chronic kidney disease	11 (6.3)	4 (4.5)	7 (8.0)
Chronic neurological disorder	12 (6.8)	9 (10.1)	3 (3.4)
Malignant neoplasm	12 (6.8)	4 (4.5)	8 (9.2)
Rheumatological disorder	9 (5.1)	5 (5.6)	4 (4.6)
Smoking			
Current	7 (4.0)	2 (2.2)	5 (5.7)
Former	50 (28.4)	30 (33.7)	20 (23.0)
Never	119 (67.6)	57 (64.0)	62 (71.3)
Laboratory values at randomization			
Leukocyte count, cells/μL			
No.	174	88	86
Median (IQR)	7000 (5200-9600)	6700 (5400-9300)	7300 (5000-10 100)
Lymphocyte count, cells/μL			
No.	174	88	86
Median (IQR)	800 (600-1200)	800 (600-1300)	800 (600-1200)
C-reactive protein, mg/dL			
No.	173	88	85
Median (IQR)	8.78 (3.70-15.20)	9.05 (3.41-15.57)	8.63 (3.97-14.94)
D-dimer, μg/mL			
No.	164	81	83
Median (IQR)	0.608 (0.397-1.014)	0.607 (0.429-0.984)	0.608 (0.375-1.030)
Ferritin, ng/mL			
No.	169	85	84
Median (IQR)	876.0 (452.5-1413.5)	976.0 (452.5-1437.5)	803.5 (443.8-1365.2)
Interleukin-6, pg/mL			
No.	114	61	53
Median (IQR)	16.5 (6.1-47.0)	16.0 (5.2-47.2)	17.0 (6.7-46.9)
Lactate dehydrogenase, U/L			
No.	171	86	85
Median (IQR)	381.0 (305.0-496.0)	389.0 (310.0-500.3)	376.0 (291.5-486.0)
Treatment at randomization			
Dexamethasone	88 (50.0)	45 (50.6)	43 (49.4)
Remdesivir	32 (18.2)	18 (20.2)	14 (16.1)
Azithromycin	20 (11.4)	7 (7.9)	13 (14.9)
Methylprednisolone	13 (7.4)	6 (6.7)	7 (8.0)
Lopinavir-ritonavir	8 (4.5)	4 (4.5)	4 (4.6)
Hydroxychloroquine	10 (5.7)	6 (6.7)	4 (4.6)

Abbreviation: SoC, standard of care.

SI conversion factors: To convert C-reactive protein to milligrams per liter, multiply by 10; D-dimer to nanomoles per liter, multiply by 5.476; ferritin to micrograms per liter, multiply by 1; lactate dehydrogenase to microkatals per liter, multiply by 0.0167; leukocytes to cells ×10^9^ per liter, multiply by 0.001; lymphocytes to cells ×10^9^ per liter, multiply by 0.001.

^a^ Percentages might not total 100% because of rounding.

^b^ Body mass index is calculated as weight in kilograms divided by height in meters squared.

^c^ Comparison of the proportion of patients requiring oxygen supplementation at baseline was significantly different between groups (*P* = .005) despite randomization.

^d^ These are the most common comorbidities observed in more than 5% of patients.

The median (IQR) daily dose of anakinra was 375 (367-375) mg, and the median (IQR) number of doses received was 60 (47-60) doses (eTable 2 in Supplement 2). Twenty-seven patients (30.3%) prematurely discontinued treatment with anakinra before 15 days. Pulse therapy with methylprednisolone was administered to 33 patients (37.1%) in the anakinra group and to 34 patients (39.1%) in the SoC group (eTable 3 in Supplement 2). Details of treatments administered for COVID-19 before and after randomization are provided in eTable 4 and eTable 5 in Supplement 2; 44 patients (24.7%) were given remdesivir and 90 (50.0%) were given dexamethasone.

### Efficacy Outcomes

**Figure 2.**
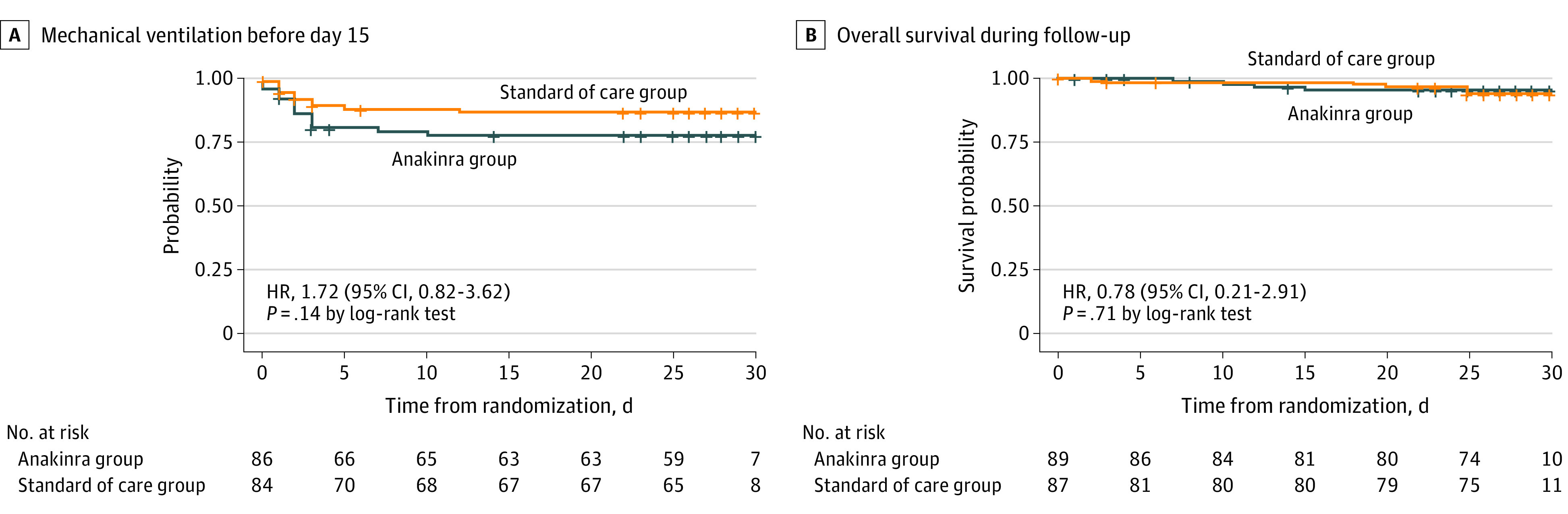
Kaplan-Meier Curves for Patients Who Received Anakinra Plus Standard of Care vs Patients Who Received Standard of Care Alone Graphs show time to mechanical ventilation until day 15 (A) and overall survival during follow-up (B). Patients were censored at the time of the last available follow-up if still alive for survival analysis. Unadjusted hazard ratios (HRs) with 95% CIs were estimated by Cox regression analyses.

The proportion of patients not requiring mechanical ventilation (noninvasive or invasive mechanical ventilation) up to day 15 in the anakinra group was not different from that in the SoC group (64 of 83 patients [77.1%] vs 67 of 78 patients [85.9%]; RR, 0.90; 95% CI 0.77-1.04; *P* = .16) (Table 2). There was no significant difference between groups in the proportion of patients not requiring invasive mechanical ventilation up to day 15 (RR, 0.99; 95% CI, 0.88-1.11; *P* > .99). Time to mechanical ventilation was not significantly different between groups (unadjusted HR for anakinra vs SoC, 1.72; 95% CI, 0.82-3.62; *P* = .14) (Figure 2A). After 15 days of treatment, the median (IQR) Po_2_/FiO_2_ ratio was 440.5 (306.9-501.2) in the anakinra group vs 270.3 (215.0-428.6) in the SoC group (eFigure 1 in Supplement 2). Data on the evolution of vital signs and respiratory function are provided in eTable 6 in Supplement 2. Although the proportion of patients requiring oxygen supplementation was not significantly different, it was numerically greater in the anakinra group than in the SoC group (RR, 1.48; 95% CI, 1.02-2.14) (Table 2).

**Table 2.** Primary and Secondary Efficacy Outcomes

Outcomes	Patients, No./total No. (%)		RR or HR (95% CI)	*P* value
	Anakinra group (n = 89)	SoC group (n = 87)		
Primary outcome: patients not requiring mechanical ventilation (invasive or noninvasive) up to day 15	64/83 (77.1)	67/78 (85.9)	RR, 0.90 (0.77-1.04)	.16^a^
Secondary outcomes				
Time to mechanical ventilation				
No.	86	84	HR, 1.72 (0.82-3.62)	.14^b^
Median (IQR)	NR	NR		
Oxygen supplementation until day 15^c^	14 (93.3)	12 (63.2)	RR, 1.48 (1.02-2.14)	.05^d^
Hospital stay, d				
No.	71	69	NA	.01^e^
Median (IQR)	12.0 (8.0-17.0)	9.0 (6.0-13.0)		
ICU admission	13/84 (15.5)	13/81 (16.0)	RR, 0.96 (0.48-1.95)	>.99
ICU stay, median (IQR), d	11 (5.0-27.5)	20 (7.0-30.0)	NA	.39^a^
Mortality at day 28	4/84 (4.8)	5/81 (6.2)	RR, 0.77 (0.21-2.77)	.74^d^
Mortality at 48 h since admission	0/84	0/81	NA	NA
Mortality at day 7 since admission	0/84	1/81 (1.1)	NA	>.99^d^
Mortality at 48 h since ICU admission	0	0	NA	NA
Mortality at day 7 since ICU admission	1/84 (1.1)	0/81	NA	NA
Negative PCR test, No. (%)	25 (46.3)	20 (43.5)	RR, 1.07 (0.69-1.65)	.84^a^
Outcomes analyzed post hoc				
Patients not requiring invasive mechanical ventilation up to day 15	73/84 (86.9)	71/81 (87.7)	RR, 0.99 (0.88-1.11)	>.99
Time to mechanical ventilation or death				
No.	86	84	HR, 1.58 (0.77-3.25)	.22
Median (IQR)	NR	NR		
Rescue medication requirement	3/89 (3.4)	16/87 (18.4)	NA	.001^a^

Abbreviations: HR, hazard ratio; ICU, intensive care unit; NR, not reached; PCR, polymerase chain reaction; RR, risk ratio; SoC, standard of care.

^a^ Calculated with χ^2 ^test.

^b^ Calculated with log-rank test.

^c^ Patients who were receiving supplemental oxygen at randomization were excluded from the analyses (74 in the anakinra group and 67 in the SoC group). Data were available for 15 patients in the anakinra group and 19 patients in the SoC group.

^d^ Calculated with Fisher exact test.

^e^ Calculated with Mann-Whitney *U* test.

The proportion of patients requiring ICU admission up to day 15 did not differ between the groups (RR, 0.96; 95% CI, 0.48-1.95) (Table 2). The length of ICU stay was not significantly different either (median [IQR], 11 [5.0-27.5] days in the anakinra group vs 20 [7.0-30.0] days in the SoC group). The viral clearance rate at day 15 did not differ between the groups (RR, 1.05; 95% CI, 1.49-0.74) (Table 2).

By day 28, 4 patients in the anakinra group had died, vs 5 patients in the SoC group (RR, 0.77; 95% CI, 0.21-2.77; *P* = .74) (Table 2). Mortality at day 28 did not differ between the groups, with a cumulative survival rate of 92.9% in the anakinra group vs 92.6% in the standard treatment group (Figure 2B). The unadjusted HR for mortality was 0.78 (95% CI, 0.21-2.91; *P* = .71) (Figure 2B).

The sensitivity analysis assessing the possible bias due to lost to follow-up patients on the primary outcome (eTable 6 in Supplement 2) suggests stability of results. More precisely, the RR for the best case scenario (ie, none of the patients lost to follow-up require mechanical ventilation) was 0.90 (95% CI, 0.78-1.03), and the RR for the worst case scenario (ie, all patients lost to follow-up required mechanical ventilation) was 0.93 (95% CI, 0.79-1.10).

### Exploratory Outcomes

Levels of relevant laboratory test values and hyperinflammatory parameters over time are shown in eTable 7 and eTable 8 respectively in Supplement 2, and are graphically represented in eFigure 1 and eFigure 2 in Supplement 2. CRP levels were significantly lower in the anakinra group vs the SoC group by day 15 (median [IQR], 1.4 [0.6-7.4] mg/dL vs 4.0 [1.3-18.1] mg/dL) (eFigure 1 in Supplement 2). Mixed-effect model results showed no significant differences between groups in the evolution of the main parameters (eTable 9 in Supplement 2).

Post hoc sensitivity analysis showed that there were no significant differences between groups for the primary and secondary end points among patients with a SCOPE score of 6 or higher (eTable 10 in Supplement 2). Post hoc Cox regression analysis showed that after adjustment by COVID-19 severity and dexamethasone use, the difference in the proportion of patients not requiring mechanical ventilation between the groups remained nonsignificant (HR, 1.84; 95% CI, 0.87-3.88) (eTable 11 in Supplement 2). The adjusted analysis yielded an HR for mortality of 1.02 (95% CI, 0.27-3.87) (eTable 12 in Supplement 2). Time to mechanical ventilation or death was not significantly different between groups (HR, 1.58; 95% CI, 0.77-3.25; *P* = .22). Likewise, no significant differences were observed in viral clearance with a post hoc adjusted multivariate logistic regression analysis (odds ratio, 0.93; 95% CI, 0.41-2.09) (eTable 13 in Supplement 2). Three patients (3.4%) in the anakinra group and 16 patients (18.4%) in the SoC group required rescue therapy (eTable 14 in Supplement 2).

Post hoc analyses showed a significantly lower proportion of patients treated with anakinra with abnormal chest imaging findings by day 15 vs the patients treated with SoC alone (28 patients [62.2%] vs 42 patients [82.4%]; RR, 0.47; 95% CI, 0.23-0.94; *P* = .04) (eTable 15 in Supplement 2). By day 28, a total of 71 patients (87.0%) in the anakinra group had been discharged from hospital vs 69 patients (86.3%) in the SoC group. By day 28, a total of 11 patients (13.6%) in the anakinra group remained in the ICU vs 13 patients (16.3%) in the SoC group. Symptoms of persistent COVID-19 at day 28 are reported in eTable 16 in Supplement 2. Time to defervescence was 2 days for the patients in the anakinra group and 3 days for the patients in the SoC group.

### Safety

The frequency of adverse events was similar in both groups (Table 3 and eTable 17 in Supplement 2). Twelve patients developed infusion-related reactions, and no patient developed neutropenia. The proportion of patients with at least 1 serious adverse event did not differ between groups. Four patients experienced at least 1 serious adverse event related to anakinra. The most common serious adverse event was respiratory failure, which was reported in 4 patients in the anakinra group and in 7 patients in the SoC group. Acute respiratory distress syndrome was recorded in 5 patients in the anakinra group and in a single patient in the SoC group. Three patients receiving anakinra experienced fatal adverse events compared with 4 patients treated with SoC.

**Table 3.** Adverse Events and Serious Adverse Events

Event category	Anakinra group (n = 89)	SoC group (n = 87)
Adverse events, No. (%)		
Patients with at least 1 adverse event	40 (44.9)	27 (31.0)
Patients with at least 1 infusion-related reaction	12 (13.5)	0
Total No. of adverse events	66	32
Patients with at least 1 adverse event related to anakinra	15 (16.9)	NA
Patients with at least 1 adverse event related to SoC	8 (9.0)	4 (4.6)
Patients with adverse event leading to death	3 (3.4)	4 (4.6)
Patients with adverse events leading to treatment discontinuation	17 (19.1)	1 (1.1)
Serious adverse events, No. (%)		
Patients with at least 1 serious adverse event	21 (23.6)	15 (17.2)
Total No. of serious adverse events	27	17
Patients with at least 1 serious adverse event related to anakinra	4 (4.5)	NA
Patients with at least 1 serious adverse event related to SoC	3 (3.4)	2 (2.3)
Type of adverse events, No. of patients^a^		
Acute respiratory distress syndrome	5	1
Respiratory failure	4	7
Dyspnea	3	3
Rash	2	0
Septic shock	0	2
Adverse events leading to death		
Acute respiratory distress syndrome	2	0
Pneumonia	1	0
Endotracheal intubation	0	1
Respiratory failure	0	1
Septic shock	0	2

Abbreviations: NA, not applicable; SoC, standard of care.

^a^ Most frequent adverse events reported in at least 2 patients are shown.

## Discussion

This randomized clinical trial was designed early in the COVID-19 pandemic to assess whether anakinra could reduce the need for mechanical ventilation and improve other key relevant outcomes in patients with severe COVID-19 pneumonia and hyperinflammation. This study failed to meet the primary efficacy end point of preventing mechanical ventilation, although improved oxygenation in terms of Po_2_/FiO_2_ was found in the anakinra group. In addition, there were no significant differences between groups for the secondary end points, including mortality. No safety concerns were identified in the patients treated with anakinra.

Our findings seem to agree with those of a Cochrane systematic review,^23^ which included results from 4 random clinical trials with IL-1 inhibitors up to November 3, 2021, and concluded that anakinra probably resulted in little or no increase in clinical improvement at day 28. Our findings also agree with the most recent update of the COVID-NMA initiative,^29^ on October 17, 2022, which suggests that anakinra probably reduces the risk of World Health Organization score of 7 or higher (ie, mechanical ventilation or death) around 60 days in hospitalized patients, but it probably results in little to no difference in the likelihood of clinical improvement around 28 days. In both cases, the certainty of the findings are low or very low.

Nevertheless, our results differ substantially from those of the double-blind, randomized, placebo-controlled, phase 3 SAVE-MORE trial,^22^ which found that treatment with anakinra was associated with significantly lower mortality and time to progression into severe respiratory failure vs placebo in hospitalized patients with moderate or severe COVID-19. Differences in patients’ profiles and procedures could partially explain the different results. The main comorbidity in our study was arterial hypertension (41.4%), but we do not know the comorbidity pattern of the patients in the SAVE-MORE trial. Patients with severe respiratory failure (Po_2_/FiO_2 _<150) were excluded from the SAVE-MORE trial,^22^ whereas in our study, all patients had severe pneumonia. The baseline levels of CRP and ferritin were notably higher in the patients included in our study and exceeded the prognostic cutoff values described in the SAVE-MORE study.^22^ The SAVE-MORE trial did have one important inclusion criteria: suPAR level of 6 ng/mL or higher. Unfortunately, at the time when the ANA-COVID-GEAS study began, the determination of suPAR was not available in Spain, and we cannot, therefore, determine suPAR levels in the patients included in our trial. We used a SCOPE score of 6 or higher as an equivalent for suPAR 6 ng/mL or higher for estimating progression to severe respiratory failure or death.^28^ In the ANA-COVID-GEAS study, 66.4% of patients presented with a SCOPE score of 6 or higher compared with 100% of patients included in SAVE-MORE trial.^22^ As for therapies, hydroxychloroquine and lopinavir-ritonavir were not administered in patients enrolled in the SAVE-MORE trial, whereas remdesivir^30^ and dexamethasone were administered more frequently (73.6% and 85.9%, respectively) compared with the ANA-COVID-GEAS study (24.7% and 50.0%, respectively).

The mean time from the date of diagnosis of SARS-CoV-2 infection to the date of randomization was 6 days. This means that many patients received a diagnosis of SARS-CoV-2 infection on an outpatient basis before being admitted to hospital and being randomized. The mean time from the date of diagnosis of interstitial pneumonia to date of randomization was 2 days. This delay could cause the patient to worsen abruptly and anakinra to be started late, when the hyperinflammation is already established.

Interestingly, our study found that a significantly lower proportion of patients in the anakinra group had abnormal chest imaging findings compared with patients in the SoC group at day 15, which might suggest a potential antifibrotic effect of the drug.^31^ However, larger studies specifically designed to evaluate this are needed.

### Strengths and Limitations

The strengths of this trial include its randomized design in a very representative sample of the Spanish population during the first waves of the COVID-19 pandemic. In addition, the baseline characteristics were generally well-balanced between groups, and the post hoc adjusted analyses yielded similar results as the unadjusted analyses regarding the risk of mechanical ventilation and death.

One of the main limitations of the study is its open-label design. It is not expected that this influenced the risk of bias in the measurement of the outcomes, because they are objective or very protocoled measures, but it could have had some influence on the deviations from the intended interventions, such as a differential use of cointerventions. The need for prohibited medication was higher in the SoC group, which may have induced an underestimation of the benefit of anakinra. Another limitation is that, although the analysis was conducted in an ITT basis, there were some missing data for principal and secondary outcomes that may be lost to follow-up for nonrandom reasons. We consider that this did not bias the results, because the percentage of missing data is not high (5%) and the sensitivity analysis for different scenarios suggests stability of the results regardless the assumptions. Another limitation is that the hospital effect has not been included in the analysis, although we consider that this has not had an important effect because we did not expect high heterogeneity among centers. Regarding randomization, the proportion of patients requiring oxygen supplementation at baseline was significantly higher in the anakinra group, but we think that it is related to chance and does not imply a bias in the randomization procedure, so no adjustment for it has been conducted. Furthermore, the sample size was not high enough to detect moderate differences between groups in primary and secondary outcomes, but we consider this information useful for future studies and meta-analyses.

## Conclusions

The findings of this randomized clinical trial suggest that the use of anakinra together with SoC does not prevent the need for mechanical ventilation or reduce mortality risk compared with SoC alone in hospitalized patients with severe COVID-19 pneumonia and hyperinflammation. Although the primary and key secondary outcomes were not met, anakinra may have a role as an early treatment for patients with less-severe disease and inflammation.

## Notes

Supplement 1.
Trial Protocol

Supplement 2. **eAppendix 1.** List of Participating Centers **eAppendix 2.** Supplemental Methods **eTable 1.** Baseline Vital Signs, Respiratory Function, and Laboratory Values **eTable 2.** Anakinra Administration and Discontinuation **eTable 3.** Methylprednisolone Pulse Therapy Administered Until Day 15 **eTable 4.** Treatments for COVID-19 Received Before and After Randomisation During the Trial **eTable 5. **Dose and Duration of Treatments for COVID-19 Received Before and After Randomisation During the Trial **eTable 6.** Sensibility Analysis Results for the Primary Outcome for Different Scenarios Regarding Assumptions of the Lost to Follow-up Patients **eTable 7.** Vital Signs and Respiratory Function by Timepoint **eTable 8.** Levels of Hyperinflammatory Parameters and Other Relevant Laboratory Parameters by Timepoint **eTable 9.** Evolution of Hyperinflammatory Parameters Over Time **eTable 10.** Primary and Secondary Efficacy Outcomes in Patients With SCOPE Score ≥ 6 **eTable 11. **Adjusted Multivariate Cox Regression Hazard Model to Estimate Risk Factors for Mechanical Ventilation **eTable 12.** Cox Regression Hazard Model to Estimate Risk Factors for Survival **eTable 13. **Logistic Regression Analysis Model of Factors Affecting Viral Clearance **eTable 14.** Rescue Therapy **eTable 15.** Imaging Assessment **eTable 16.** Symptoms of COVID-19 at Day 28 **eTable 17.** Serious Adverse Events **eFigure 1.** Evolution of the First Panel of Exploratory Outcomes **eFigure 2.** Evolution of the Second Panel of Exploratory Outcomes
eReferences

Supplement 3.
GEAS-SEMI Nonauthor Collaborators

Supplement 4.
Data Sharing Statement
