# Supplementary material for: Efficacy and Safety of Anakinra Plus Standard of Care for Patients With Severe COVID-19: A Randomized Phase 2/3 Clinical Trial
Source: JAMA Netw Open. 2023 Apr 7;6(4):e237243. doi: 10.1001/jamanetworkopen.2023.7243 (PMC10082404; doi:10.1001/jamanetworkopen.2023.7243)
Supplement: Supplement 1. — Trial Protocol [file jamanetwopen-e237243-s001.pdf]

CLINICAL TRIAL OF THE USE OF ANAKINRA (ANTI IL-1) IN CYTOKINE STORM SYNDROME (CSS) SECONDARY TO COVID-19

A phase 2/3, randomized, open-label, parallel group, 2-arm, multicenter study investigating the efficacy and safety of intravenous administrations of anakinra, an interleukin-1(IL-1) receptor antagonist, added to standard of care, versus standard of care, in reducing hyper-inflammation and respiratory distress in patients with SARS- CoV-2 infection.

Protocol Number: ANA-COVID-GEAS

EudraCT Number: 2020-001825-29

Type of Study: Phase 2/3

Sponsor: Navarrabiomed – Fundación Miguel Servet
C/Irunlarrea nº3, 31008 Pamplona. Tlf: 848 422 163

Signature Date September 30th 2020

Principal Coordinating Investigator: Dra. Patricia Fanlo Mateo
Servicio de Medicina Interna - Complejo Hospitalario de Navarra

Signature Date September 30th 2020

Index

1 Ethics 4
1.1 Independent ethics committee 4
1.2 Ethical conduct of the study 4
1.3 Patient information and consent 4
1.4 Data protection 5
2 Introduction 6
2.1 Background 6
2.2 Study rationale 8
2.3 Potential risks and benefits 9
3 Study objectives and endpoints 10
3.1 Primary objective 10
3.2 Secondary objective 10
3.3 Exploratory objective 11
4 Investigational plan 12
4.1 Overall study design and plan 12
4.2 Discussion of study design, including the choice of control groups 13
4.3 Selection of study population 13
4.4 Treatments 16
4.5 Efficacy and safety assessments 21
5 Quality control and quality assurance 33
6 Statistical plan 34
6.1 Determination of sample size 34
6.2 Definition of study populations 35
6.3 Overall statistical and analytical plan 35

7 Data collection, handling and record keeping......... 39

7.1 Data standards 39

7.2 Case report form 39

7.3 Source data 39

7.4 Protocol deviations 40

7.5 Database closure 40

7.6 Record retention 40

8 End of study 42

9 Sponsor's discontinuation criteria 43

10 Dissemination and publication of results 44

11 Reference list 45

12 Anexo 48

1 Ethics

1.1 Independent ethics committee

It is the responsibility of the investigator to obtain approval of the study protocol, possible amendments and the written subject information and informed consent form from the IEC. The investigator should file all correspondence with the IEC.

1.2 Ethical conduct of the study

This study will be conducted in compliance with this protocol, applicable regulatory requirements, and in accordance with the ethical principles that have their origin in the Declaration of Helsinki).

1.3 Patient information and consent

Given the special epidemiological circumstances in which we find ourselves, we consider that the paper record of informed consent suggests additional risks of transmission of the COVID-19. For this reason, the protocol will be explained to the patient in front of a nurse who will act as a legal witness by signing the document on behalf of the patient.

Procedure:

1. Explain to the patient that he/she is proposed to participate in the study; his/her participation in the study is voluntary, his/her attendance will be carried out according to the usual protocols in force at each moment with the addition of anakinra or placebo according to the randomization.
2. Be informed of any procedures in addition to the usual care provided by the study protocol.
3. You will be informed that the study entails the inclusion of your anonymized clinical data in a database with the legally established levels of protection.
4. You will be informed that you have the right to withdraw from the study at any time, without giving any reason for doing so and without your medical care being affected.

5. You will be expressly asked if you agree to participate in the study and your answer will be recorded in the note on the evolution of the EHR, with the expression the patient agrees to participate in the study

1.4 Data protection

1. Participants will be assigned a unique identifier by the investigator. Any participant records or datasets that are transferred to the sponsor will contain the identifier only; participant names or any information which would make the participant identifiable will not be transferred.
2. The participant must be informed that his/her personal study-related data will be used by the sponsor in accordance with local data protection law. The level of disclosure must also be explained to the participant.
3. The participant must be informed that his/her medical records may be examined by Clinical Quality Assurance auditors or other authorized personnel appointed by the sponsor, by appropriate IRB/IEC members, and by inspectors from regulatory authorities.

2 Introduction

2.1 Background

This protocol has been prepared by the Systemic Autoimmune Diseases Group (GEAS) steering committee from the Spanish Society of Internal Medicine (SEMI) in response to the current urgent situation that we are living in Spain because of the COVID-19 pandemic. The GEAS member are experts in the treatment of autoinflammatory diseases and the use of biologic drugs in general, and anakinra in particular. importantly, in Spain, internal medicine physicians are currently involved in the treatment of patients infected with SARS-CoV-2.

The objective of this study is to investigate new possibilities to reduce the number of patients requiring mechanical ventilation. This is intended to address the most urgent need to preserve the access to intense care unit support to the lower possible number of patients and may potentially reduce mortality.

In December 2019, a respiratory condition caused by what was later identified as a new coronavirus (SARS-CoV-2) was detected in Wuhan Province, China (1). In these four months, the virus has spread throughout the world's population, in more than 100 countries and affecting thousands of people. The WHO declared in March that we were facing a pandemic because of this virus, in which Europe could now be considered the epicenter. The strict containment measures implemented in China, the mass detection of the virus with subsequent isolation of cases and contacts that has been carried out in countries such as Singapore, Taiwan or South Korea seem to have been useful in limiting the virus´ spread. Consequently, in our country, and given the extension of the infection, a state of alarm has been decreed, at least for four weeks. Despite the containment measures, numerous cases are still being diagnosed every day, with an approximate lethality of 3.7%, especially in those considered at risk such as patients over 60 years of age and with comorbidities (Report nº 17. COVID-19 situation in Spain on 27 March 2020. COVID-19 team. SiViES. CNE. CNM. ISCIII).

When SARS-CoV-2 infects the respiratory tract, it causes the disease called COVID-19. From the point of view of the natural evolution of the disease, this can be a mild respiratory syndrome, which will occur in approximately 80% of cases, or a more serious disease, with the appearance of pulmonary infiltrates and in some patients a respiratory distress with rapidly progressive worsening (2). This second phase, which is only reached by a subgroup of

patients, is usually seen in the interval between day 7 and day 10 of the infection from the onset of symptoms. So far, there is no proven explanation for this great variability in its clinical expression.

The treatment used in our country so far is based on the combination of hydroxychloroquine, azithromycin and antiretrovirals (protease inhibitors, lopinavir), based on previous experiences. Several clinical trials are currently underway in Spain: PanCOVID-19 (Azithromycin + Hydroxychloroquine + lopinavir/ritonavir), GS-US-540-5774 (Remdesivir), EFC16844 (Sarilumab) and WA42380 (Tocilizumab) as well as international projects such as Discovery (NCT04315948), Solidarity and REMAP-CAP which include other treatment alternatives such as interferon-beta-1B or Anakinra.

According to recent data from the Chinese cohorts, a subgroup of patients with COVID-19 shows a very marked increase in inflammatory test data, such as lymphopenia, thrombocytopenia, C-reactive protein (CRP) levels, lactate dehydrogenase (LDH), IL6 (> 40 pg / mL) and D-dimer (> 0.28 μg / L), the latter two being predictive of the development of severe pneumonia (sensitivity 93.3%, specificity 96.4%) (2,3,4). Accumulating evidence suggests that a subgroup of patients with severe COVID-19 might have a cytokine storm syndrome (CSS). In this group of patients in which this "inflammatory profile" is detected, the damage observed is not a direct consequence of the viral infection (viral necrotizing pneumonia) but rather a secondary hyperimmune response, mainly related to the monocyte/macrophage activation, which has also been observed in autopsy studies and previously in other infections by other types of coronavirus (5,6). This CSS is superimoosable to that observed in patients with secondary hemophagocytic lymphohistiocytosis (sHLH), often triggered by viral infections or macrophage activation syndrome (MAS), secondary to systemic and autoinflammatory autoimmune diseases.

In this group of patients there is a significant release of pro-inflammatory cytokines, including interleukin (IL) -1β and IL-6 (6). Of these, IL-1β has a greater pathogenic relevance, given that it promotes the inflammatory cascade and also induces the synthesis of several inflammatory genes such as IL-6 itself, IL-8, MCP-1, COX-2, IκBα, IL-1α, IL-1β and MKP-1. Numerous case reports and case series have been published supporting the use of IL-1β blockade in the MAS (8-12). At this time, there is some experience in the treatment of COVID-19 in the subgroup of patients in the inflammatory phase with Tocilizumab (IL-6 receptor blocker), but

no controlled studies have been completed as yet. However, IL-6 is not a key molecule in the cytokine storm that triggers MAS (9).

Anakinra (IL-1Ra) is a recombinant IL-1 receptor antagonist with a very short half-life of 4-6 hours, requiring a daily subcutaneous injection of a 100 mg dose. It has also been shown to reduce levels of other pro-inflammatory cytokines (including IL-6 and IL-18) and acute phase reactants such as PCR and ferritin. Therefore, it has been used in the control of autoinflammatory syndromes and in patients with MAS (7,8). In addition, data have been reported from a phase 3 randomized controlled trial of IL-1 blockade with anakinra in sepsis with characteristics of MAS, which showed a significant improvement in the 28-day survival rate (65.4% anakinra vs. 35.3% placebo), with HR for death 0.28 (0.11-0.71, $p = 0.0071$), with no increased adverse events (11).

2.2 Study rationale

As shown by the data available in the most recent literature generated from the Chinese experience, and by the most recent data made available by the different Spanish hospitals responsible for the management of these patients, hyper- inflammation, caused by a cytokine storm resulting from an exaggerated response of the immune system to the presence of the virus (CSS-like pneumonia), is considered to represent one of the most important negative prognostic factor in patients infected with SARS-CoV-2. The inclusion criteria for COVID-19 used in this protocol is based on the analysis of routine blood chemistry data obtained from patients with SARS-CoV-2 infection. This criterion has been designed with high sensitivity (>90%) for patients who require ICU admission (12). This constitute the rationale for testing drugs specifically targeted to reduce the cytokine storm.

This protocol has been prepared for the purpose of addressing the current medical emergency, given the severity of the disease and the extremely high number of individuals affected. The objective of this study is to investigate anakinra to reduce the number of patients requiring mechanical ventilation. This is intended to address the most urgent need to preserve the access to intense care unit support to the lower possible number of patients and may potentially reduce mortality.

2.3 Potential risks and benefits

The potential applicability of the results of this clinical trial, in an extraordinary health emergency such as COVID-19, is obvious. Positive results could help to define the standard treatment of severe COVID-19 pneumonia and have a dramatic impact on both local and global morbidity and mortality. Negative results would constitute very useful scientific evidence for abandoning unfounded empirical approaches and would help redirect resources and efforts in another more promising direction. The expected length of time needed for recruitment is 3-4 weeks, depending on the epidemiological progression of the pandemic in our centers, so results could be available in early May 2020.

Anakinra is a recombinant form of the human IL-Ra, r-metHuIL-1Ra, which is produced by recombinant DNA technology in an E. coli expression system. Therapeutically, anakinra neutralizes the biological activity of IL-1 (IL-1α and IL-1β) by competitively inhibiting its binding to the IL-1RI.

Kineret (anakinra) was first approved for treatment of RA in the US in 2001 and subsequently in the EU/EEA in 2002. In 2012, an sBLA on anakinra for treatment of NOMID was approved in the US. Kineret is also approved for treatment of CAPS (in EU/EEA, Israel and Australia), Still's disease, including SJIA and AOSD (in EU/EEA) and SJIA (in Australia).

The initial IND for anakinra was granted in 1991. The estimated cumulative exposure to anakinra in completed company sponsored clinical studies up to 1 May 2018 is 6404 subject-years, in 8518 subjects with various indications. Anakinra is administered s.c. at doses of 100 mg/day (RA) or in weight-based doses of up to 8 mg/kg/day (NOMID). In clinical studies in sepsis, doses up to 2 mg/kg/hour i.v. over 72 hours were administered to >500 patients and were well tolerated. For additional information of i.v. administration of anakinra, see Section 5.4.4.1.

3 Study objectives and endpoints

3.1 Primary objective

The primary objective of this study is to assess the effect of anakinra in addition to standard treatment on the need for mechanical ventilation in patients with severe COVID-19 and CSS pneumonia.

3.1.1 Primary endpoint

Treatment success, defined as number of patients not requiring mechanical ventilation by Day 15.

3.1.2 Secondary endpoints supporting the primary objective

- Number of patients not requiring mechanical ventilation (day 28).
- Time to mechanical ventilation (days)
- Time to oxygen saturation normalization
- Stay in ICU and hospitalization (days)

3.2 Secondary objective

The secondary objective of this study is to assess the effect of anakinra in addition to standard treatment on mortality in patients with severe COVID-19 and CSS pneumonia.

3.2.1 Secondary endpoints supporting the secondary objective

- Total mortality rate (day 28)
- Mortality 48 hours, 7 days, in ICU and hospital
- Viral clearance / viral shedding
- Frequency and severity of AEs:
    - Treatment-emergent severe fatal and life-threatening serious adverse events (SAEs).
    - Adverse events leading to premature discontinuation of study treatment.
    - Anaphylactic/anaphylactoid reactions.

- Anakinra treatment group: Severe neutropenia.
- Treatment-emergent laboratory abnormalities.

3.3 Exploratory objective

To assess the effect of anakinra on IL-6, ferritin, and selected biomarkers relevant for hyperinflammation, MAS and cytokine storm change from baseline.

3.3.1 Exploratory endpoints

- Change (improvement) in IL-6, ferritin, D-dimer, TGs, lymphopenia, CRP, ESR, LDH.
- Time to defervescence (fever end)

4 Investigational plan

4.1 Overall study design and plan

This is a randomized, open-label, parallel group, 2-arm multicenter study to investigate the efficacy and safety of anakinra in reducing hyper-inflammation and respiratory distress in adult patients with severe COVID-19 and CSS pneumonia.

The study consists of screening, a 2-week treatment period and a 2-week follow-up period.

The 2-week treatment period is open, and the patients will be randomized to treatment with anakinra + standard of care or standard of care alone in a 1:1 ratio.

Anakinra will be administered as 4-times daily i.v. infusions for a maximum of 15 days (Days 1 to 15), according to the patient health status and expert clinical opinion. The primary endpoint will be evaluated at Day 15.

According to hospital internal protocol, if the patient improves enough to recommend his/her discharge before the 15th day of the study, the patient will be able to continue receiving the medication at home by s.c. administration (100mg/6 hours). In any case, follow-up visits by telephone or in person will be made, depending on where the patient is, until the end of the study.

The study duration for an individual patient will not exceed 4 weeks. The end of the study is defined as last patient last follow-up visit/phone call.

Figure 1:

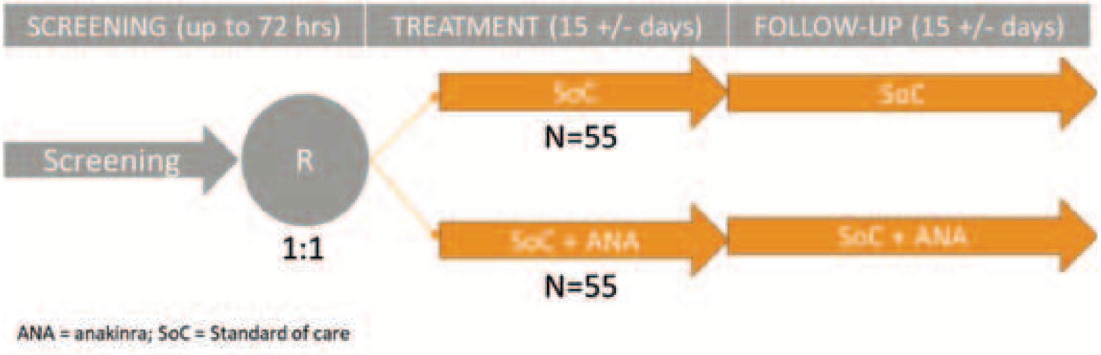

The study design has a total sample size of 180 patients and consists of two stages. At the end of the treatment stage, the success rates are compared between each arm: anakinra + standard of care treatment vs standard of care alone.

A data review committee composed of experts in internal medicine will be involved in the study oversight and interpretation of the study results.

4.2 Discussion of study design, including the choice of control groups

4.3 Selection of study population

4.3.1 Inclusion criteria

A patient must fulfill the following criteria in order to be included in the study:

- Age 18-80 years.
- Severe pneumonia COVID-19 defined as:
  - Nasopharyngeal smear with RCP positive for SARS-CoV-2
  - X-Rays (or other technique) pulmonary infiltrates compatible with pneumonia.
  - 1 or more of the following criteria:
    - Ambient air oxygen saturation <= 94% measured with a pulse oximeter.
    - Pa:FiO2 (partial pressure O2/fraction of inspired O2) <=300.
    - Sa:FiO2 (O2 saturation measured with pulse oximeter/ fraction of inspired O2) <=350.
- High suspicion of CSS that could resemble MAS-like: represented by IL-6 values > 40 pg/mL and/or ferritin >500 ug/L and/or PCR > 30 mg/L (rationale: ≥ 5 upper normal limit) and/or LDH >300 UI/L. We have chosen these parameters because they are implemented in all the participating hospitals, they are a reflection of the cytokine storm and they have also been significant in terms of predicting mortality in patients with COVID-19 (9).
- Written informed consent. The protocol will be explained to the patient in front of a nurse who will act as a legal witness by signing the document on behalf of the patient.

4.3.2 Exclusion criteria

The presence of any of the following will exclude a patient from inclusion in the study:

- Need for oro-tracheal intubation and/or invasive mechanical ventilation at the start of the study.
- AST/ALT with values greater than 5 times normal levels.
- Neutrophils < 1.500 cell/mmc.
- Platelets < 50.000 cell/mmc.
- Sepsis or pneumonia documented by other pathogens than SARS-CoV-2.
- Existence of any life-threatening comorbidity or any other medical condition that, in the investigator's opinion, makes the patient unsuitable for inclusion.
- Inability to obtain informed consent.
- Positivity for HBV or HCV.
- Patient with active tuberculosis (It is at the discretion of the researcher to perform the tuberculin test on the patient in the screening period if it is considered a risk population and on patients with active tuberculosis manifestations).
- Pregnancy.
- Use of other previous or concomitant biological treatments. Patients in concomitant treatment with other biologicals that may interfere will be excluded: tocilizumab, canakinumab, TNFalfa inhibitors, JAKiinibs
- Severe renal dysfunction (estimated glomerular filtration rate ≤ 30 ml / min / 1.73 m2) or receive continuous renal replacement therapy, hemodialysis or peritoneal dialysis.
- Uncontrolled hypertension (sitting systolic blood pressure > 180 mmHg or diastolic blood pressure > 110 mmHg).
- Administration of plasma from convalescent patients who have recovered from SARS-CoV-2 infection.
- History of hypersensitivity or allergy to any component of the study drug.
- Enrollment in another concurrent intervention clinical trial, or intake of an investigational medication within three months or 5 half-lives prior to inclusion in this study, if deemed to interfere with the objectives of this study as assessed by the investigator.

- Predictable inability to cooperate with given instructions or study procedures.

4.3.3 Withdrawal of patients from treatment or study

4.3.3.1 Withdrawal from treatment

A patient should be withdrawn from the study treatment if, in the opinion of the Investigator, it is medically necessary, or if it is the wish of the patient.

When a patient is withdrawn, the date of last IMP (investigational medicinal products) administration and the date and reason for treatment withdrawal should be clearly described in the relevant sections of the CRF. If a patient is removed from treatment because of an AE, the reason for treatment withdrawal should always be stated as 'adverse event' irrespective of whether this was the investigator's or the patient's decision.

If a patient withdraws due to worsening of disease, patient should be treated as a non-responder. In these case, the patient will be able to receive treatments that are protocolized at their hospital. The patient could be treated with pulses of steroids, intravenous inmunoglobulins, betainterferon and other biologic or immunosuppressants agents such as cyclosporin, tacrolimus, baricitinib , tocilizumab, sarilumab , ruxolitinib…

The patient will continue to participate in the study without taking study treatment.

4.3.3.2 Withdrawal from study

Whenever possible and irrespective of the reason for withdrawal, the patient should be examined as soon as possible. Relevant samples should be obtained, and all relevant assessments should be completed. The CRF should be completed as far as possible. Date and reason for the study withdrawal should be clearly described in the CRF.

4.3.4 Replacement of withdrawn patients

Patients withdrawn from the study will not be replaced.

4.3.5 Screening failures

Screening failures are defined as patients who consent to participate in the study but are not subsequently randomized into the study. A minimal set of screening failure information is required which includes demographics, reason for screen failure and failed eligibility criteria.

Patients who do not meet the criteria for participation in this study (screening failure) may not be rescreened.

4.4 Treatments

4.4.1 Treatments administered

According to the randomization schedule, patients in arm A will receive (IMP) and standard of care and patients in arm B will receive standard of care only, see Table 1.

The treatment standard of care will be based on hydroxychloroquine and/or lopinavir-ritonavir and/or azithromycin.

Table 1:

Arm	Investigational product	Dosage form	Route	Daily dose	Dosage regimen
A	Anakinra	Solution	i.v. infusion	Days 1-15: 400 mg/day in total, divided into 4 doses given every 6 hours	4 times daily for 15 days
A	Hydroxychloroquine	Tablets	Oral	Day 1-15: 400 mg/day in total, divided into 2 doses given every 12hours (loading dose: 400 mg/12 h)	2 times daily for 5 days
A	Lopinavir-Ritonavir	Tablets	Oral	Day1-15: 800/200 mg/day in total, divided into 2 doses given every 12 h	2 times daily for 5-7 days.
A	Azythromycin	Tablets	Oral	Day 1-15: 500 mg /day	1 time daily for 5 days.
B	Hydroxychloroquine	Tablets	Oral	Day 1-15: 400 mg/day in total, divided into 2 doses given every 12 hours (loading dose: 400 mg/12 h)	2 times daily for 5 days
B	Lopinavir-Ritonavir	Tablets	Oral	Day1-15: 800/200 mg/day in total, divided into 2 doses given every 12 hours	2 times daily for 5-7 days.
B	Azythromycin	Tablets	Oral	Day 1-15: 500 mg /day	1 time daily for 5 days.

4.4.2 Identity of investigational medicinal products

Possible deficiencies related to the handling and quality of the IMP should be reported in the CRF and also directly to complaints@sobi.com.

4.4.2.1 Anakinra

The IMP anakinra is delivered as a sterile solution for injection, prefilled in a single-use syringe with the strength 100 mg. The total volume of injection is 0.67 mL and the concentration of anakinra in the solution is 150 mg/mL.

Anakinra must be stored at refrigerated conditions at 2-8 °C (36°-46°F) in a secure area at the study sites. Further instructions for handling and storage of the IMP anakinra are available in the IMP manual.

SOBI, will provide the study drug as well as the packing and distribution. Labeling will comply with national regulatory requirements.

4.4.3 Method of assigning patients to treatment groups

The different treatment groups are;

- Arm A: Anakinra as add-on to standard of care
- Arm B: Standard of care only

The ratio between the treatment groups is 1:1, i.e., the same number of patients will be randomized to anakinra or standard of care.

In order to assign each patient to each of the treatment branches and ensure a balance between them for the different centers, the method of random allocation by permuted blocks of sizes 10 and 12 will be used, using the randomizer library of the R statistical package, version 3.4.0.

Although the study is open, measures are taken to prevent a selection bias: one collaborator from each center will be in charge of the allocation, for which he will receive the identifying information of each recruited subject (trial identification number), attaching the branch indicated by the random procedure and returning this information to the people in charge of continuing the development of the intervention.

4.4.4 Selection of doses

4.4.4.1 Dose selection rationale for anakinra

Anakinra is approved for the chronic treatment of a number of inflammatory diseases as a subcutaneous treatment (at doses of 100 mg/day or in weight-based doses of up to 8 mg/kg/day). The i.v. administration of anakinra has been studied in clinical trials in healthy volunteers and in critically ill patients with sepsis and hyper-inflammation at variable i.v. doses up to 3500 mg/day over 72 hours: e.g., 2 mg/kg/hour, 20 mg/kg/day (<40 kg) and 916 mg/day (> 40kg), bolus of 100 mg followed by infusion of 2 mg/kg/hour. No safety concerns emerged in these studies [(14-18)].

A study in children with systemic onset juvenile arthritis complicated by refractory macrophage activation syndrome is currently ongoing (NCT02780583) in which anakinra is administered at dose of 10 mg/kg/day to a maximum of dose of 200 mg/day divided every 12 hours (for children ≤40 kg) or 5 mg/kg/day up to a maximum dose of 400 mg/day divided every 6 hours (children > 40 kg and adults).

Based on the above, in this study anakinra is administered at a total dose of 400 mg per day, divided in 4 i.v. doses of 100 mg every 6 hours.

4.4.4.2 Rationale for the use of glucocorticoids

Interim WHO guidance argues against the use of glucocorticoids in the treatment of patients with SARS-CoV-2 infection. "They should be avoided, because of the potential for prolonging viral replication as observed in MERS-CoV patients, unless indicated for other reasons" (https://www.cdc.gov/coronavirus/2019-ncov/hcp/clinical-guidance-management patients.html). However, evidence demonstrating a positive effect or a detrimental effect of glucocorticoids specifically in the treatment of patients with SARS-CoV-2 infection is lacking. In real life, glucocorticoids are being widely used in the treatment of patients in China (6).

For SARS-CoV-2 infection, a growing body of evidence shows that hyper-inflammation appears to be particularly prominent in patients with severe disease, as well as in non-survivors compared to survivors (20, 21). All together these results point to high ferritin, high D-dimers and high LDH levels as being associated with poor outcome. Furthermore, initial data show the presence of hyperproduction of inflammatory cytokines with a profile similar to that present in patients with secondary hemophagocytic lymphohistiocytosis (sHLH) (20).

As glucocorticoids are one of the mainstays of first line anti-inflammatory treatment in sHLH, they have been suggested as one of the potential treatments for patients with severe SARS-CoV- 2 infection (22). Further to the relevance of the hyper-production of inflammatory cytokines in this infection, initial promising data have been reported with the use of IL-6 targeted therapies, namely tocilizumab, in the treatment of patients with SARS-CoV-2 infection (23). Noteworthy, in this report all patients treated with tocilizumab also received glucocorticoids.

As this study is testing the hypothesis of the efficacy and safety of cytokine-targeted anti-inflammatory treatments in patients with SARS-CoV-2 infection and hyper-inflammation, it appears useful to evaluate these anti-inflammatory treatments with a background of glucocorticoids. Indeed, in sHLH, anakinra is used with a background treatment of glucocorticoids (24, 25, and 26).

A recent paper has reported the efficacy of a therapeutic approach consisting of intravenous continuous anakinra infusions in treating severely ill adult patients with sHLH/MAS. Continuous infusion of i.v. anakinra may result in rapid serologic and subsequent clinical improvement in adult patients with MAS. This method for treating cytokine storm should be considered in the current COVID-19 pandemic in the subgroup of patients with severe disease that have a cytokine storm presentation (27).

Based on anakinra benefit published by Adam Monteagudo et al. (27), a course of 1 pulse of i.v. methyrlprednisolone for 3 consecutive days (pulse dose recommendend 250-500 mg) should be considered under investigators clinical criteria in both arms at anytime during the study .

4.4.5 Selection and timing of doses for each patient

4.4.5.1 Anakinra

Anakinra will be administered by i.v. infusion at total dose of 400 mg per day, divided in 4 doses 100 mg i.v. every 6 hours.

Anakinra treatment will continue for a maximum of 15 days (Days 1 to 15) if needed, according to clinical response and HCP prescription. The date and time of each anakinra administration will be recorded in the CRF.

Before administration, the full content of the prefilled, single-use syringe (anakinra 100 mg) will be diluted in 10 mL saline. The i.v. administration of anakinra must occur immediately after the preparation over an infusion period of 5 minutes.

Full instructions for the preparation of anakinra are available in the IMP manual.

4.4.6 Blinding and unblinding

This is an open study with no level of blinding.

4.4.7 Concomitant therapy

4.4.7.1 Background therapy

A course of 1 pulse of IV methyrlprednisolone for 3 consecutive days (pulse dose recommended 250-500 mg) should be considered under investigators clinical criteria in both arms at any time during the study .

4.4.7.2 Concomitant therapy

Concomitant use of tocilizumab, canakinumab, TNF inhibitors and JAK inhibitors is not allowed.

Antimicrobial therapy and prophylaxis are not limited.

Analgesic treatment, transfusion of blood products, electrolyte and glucose infusions, i.v. parenteral nutrition, inotropic support, antibiotics, anti-fungal and anti-viral treatments, ultrafiltration or hemodialysis, as well as general supportive care are permitted.

Other therapy considered necessary for the patient's welfare may be given at the discretion of the Investigator.

All relevant concomitant therapy, as defined by the Investigator, will be recorded in the CRF.

4.4.8 Treatment compliance

The IMP administrations will be recorded in the CRF.

Product accountability records will be kept. The pharmacy and Investigator must maintain accurate records demonstrating date and amount of study drug(s) received, to whom and by whom administered or dispensed (patient-by-patient accounting).

4.4.9 Rescue Medication

All patients must have access to rescue treatment with independent of the assigned arm.

If a patient withdraws due to worsening of disease, patient should be treated as a non-responder.

In the case of a patients starts to worsening independent of the assigned arm, , the patient will be able to receive treatments that are protocolized at their hospital. The patient could be treated with pulses of steroids, intravenous inmunoglobulins, betainterferon , immunosuppressants agents such as cyclosporin, tacrolimus and other biologic agents such as baricitinib , tocilizumab, sarilumab and ruxolitinib. If the patient need therapy with biological drugs they should be removed from the study.

4.5 Efficacy and safety assessments

4.5.1 Study schedule

4.5.1.1 Schedule of events

Measurements will be done during screening (before Day 1), at Day 1, Day 4, Day 7, and Day 15 and Day 28.

Table 3: Clinical and laboratory parameters to be collected

ASSESSMENT	SCREENING	TREATMENT PERIOD				Follow-up
	Prior to Visit 1	Visit 1	Visit 2	Visit 3	Visit 4	Visit 5/TC
		Day 1 (Baseline)	Day 4	Day 7	Day 15 (± 2 day)	Day 28 (± 2 days)
Informed consent	X					
Eligibility criteria	X	X				
Patient information	X					
Physical examination	X					
Vital signs assessment	X	X	X	X	X	
EKG assessment	X				X	
Chest X-Ray	X				X	

Laboratory assessments (local)	X	X	X	X	X	
Urine pregnancy test	X				X	
Randomization		X				
IMP administration		X X				
Concomitant medication incl background therapy		X	X	X	X	
Pulmonary function		X	X	X	X	
PaO_2/FiO_2	X				X	
Resting SpO_2 (3 times per day)		X	X	X	X	
Oxygen supplementation		X	X	X	X	X
MEWS score		X			X	
Survival						X
Time to hospital discharge						X
Adverse events		X	X	X	X	
Biomarkers		X	X	X	X	

4.5.1.2 Screening

Screening will occur prior to Visit 1 (Baseline). The aim of the screening is to collect necessary data to confirm the patient's eligibility and can be ongoing up the randomization of the patient at Visit 1. A signed informed consent form must be obtained from the patient prior to any study-related activities. However, laboratory results already available in medical records at the time of informed consent can be used to confirm eligibility, if collected prior to Visit 1. Please refer to Table 3 for the clinical and laboratory parameters to be collected during screening.

Patients who do not meet the criteria for participation in this study (screening failure) may not be rescreened.

4.5.1.3 Visit 1, Baseline (Day 1)

Once all inclusion and exclusion criteria have been reviewed and recorded, and the patient has been found eligible, the patient will be randomized into one of the treatment groups and the first dose of study drug will be administered accordingly (see Section 4.4.1). Please refer to

Table 3 (see Section 4.5.1), for other clinical and laboratory parameters to be collected during the visit.

During the treatment period, clinical and laboratory assessments and procedures should preferably be performed before IMP administration.

4.5.1.4 Visits 2 to 3 (Days 4 and 7)

Please refer to Table 3 for the clinical and laboratory parameters to be collected during these visits. For study drug administration schedule, see Section 4.4.1.

Effort should be made to adhere to the schedule of assessment. However, a window of ±1 day will be allowed for all assessments scheduled. This does not include IMP administration.

During the treatment period, clinical and laboratory assessments and procedures should preferably be performed before IMP administration.

4.5.1.5 Visit 4 (Day 15)

Please refer to Table 3 for the clinical and laboratory parameters to be collected during these visits. For study drug administration schedule, see Section 4.4.1.

Effort should be made to adhere to the schedule of assessment. However, a visit window of ±2 day will be allowed for Day 15.

During the treatment period (Days 1 to 15), clinical and laboratory assessments and procedures should preferably be performed before IMP administration.

The primary endpoint will be evaluated at Day 15.

4.5.1.6 Follow-up (Day 28)

These visits can be conducted either as a follow-up telephone call or in-person visit, if the patients is still hospitalized. Please refer to Table 3 for the clinical parameters to be collected during the visits.

A window of ±5 days will be allowed.

4.5.2 Patient information and physical examination

4.5.2.1 Demographics

The patient´s date of birth and gender will be collected at screening and recorded in the CRF.

4.5.2.2 Medical and surgical history

Details of the patient's relevant medical and surgical history as judged by the Investigator will be collected at screening and recorded in the CRF.

4.5.2.3 Prior medication

Details of the patient's relevant prior medication as judged by the Investigator will be collected at screening and recorded in the CRF.

4.5.2.4 Physical examination

A general physical examination will be performed at screening and recorded in the CRF. The assessment will be reported as "normal" or "abnormal". Any abnormalities should be specified and recorded as medical history.

Body weight and height will be recorded as a part of the physical examination.

4.5.3 Efficacy assessments

4.5.3.1 Pulmonary function (primary efficacy assessment)

The date and time of the requirement for mechanical ventilation will be assessed at Visits 1 to 4, and recorded in the CRF.

4.5.3.2 MEWS score

Modified early warning system score (MEWS) will be assessed at Day 1 and Day 15 and recorded in the CRF if ICU is needed.

4.5.3.3 SpO2

Resting peripheral capillary oxygen saturation (SpO2) will be measured 3 times per day by pulse oxymetry at Visits 1 to 4 and recorded in the CRF. Saturation normalization will be measured, both as an absolute value and as a relative value with respect to the baseline situation of income.

4.5.3.4 *Oxygen supplementation*

Oxygen supplementation will be assessed at Visits 1 to 5. The date, time and amount of supplementation will be recorded in the CRF.

4.5.3.5 *Thorax radiography*

A thorax radiography (Rx) will be performed at screening and Visit 4 (Day 15). The Rx scan will be recorded in the CRF and reported as "normal" or "abnormal".

Any abnormalities reported at screening should be specified and recorded as medical history.

4.5.3.6 *Laborarατory assessments*

Blood samples for determination of the laboratory assessments described in Table 4 will be drawn at Visits 1 to 4. The date and time of blood sampling will be recorded in the CRF.

Table 4 Laboratory assessments:

**Biochemistry**	**Hematology**
Aspartate aminotransferase (AST)	Hemoglobin
Alanine aminotransferase (ALT)	ESR
Total bilirubin (if >upper limit of normal also conjugated and non-conjugated bilirubin)	Platelet count
Prothrombin Time/International Normalized Ratio (PT-INR)	White blood cells
Fibrinogen	RBC
Cardiac troponin	Differential blood count
Creatinine	**Hyperinflammatory parameters**
C-reactive protein (CRP)	Ferritin
	LDH
	D-dimers
	IL-6

All laboratory samples will be analyzed at the respective local hospital laboratory according to their standard routines.

4.5.3.7 *Survival and hospital discharge*

Data on survival and hospital discharge will be collected at Visits 5 and 6 and recorded in the CRF.

4.5.4 Safety assessments

4.5.4.1 Adverse events

Adverse event (AE)

The safety profile of anakinra has been consistent across indications, age groups, and doses studied; this includes data that have been reported in the literature for off-label indications. The safety profile has remained stable also when anakinra has been studied in long-term safety studies and in patients with various co-morbidities. There are no indications of increasing AE rates over time. The most common AEs are non-serious, mostly mild to moderate injection site reaction that usually occur early and resolve during continued anakinra treatment.

An AE is any adverse change, i.e., any unfavorable and unintended sign, including an abnormal laboratory finding, symptom, or disease that occurs in a patient during the course of the study, whether or not considered by the investigator as related to study treatment.

All AEs for the patients that have received anakinra should be reported irrespective of causality to meet regulatory requirements. The sponsor of the study is responsible for expedited reporting as per regulatory requirement.

A treatment-emergent AE is any AE temporally associated with the use of study treatment whether or not considered by the investigator as related to study treatment.

Adverse events include:

- Exacerbation of a pre-existing disease.
- Increase in frequency or intensity of a pre-existing episodic disease or medical condition.
- Disease or medical condition detected or diagnosed during the course of the study even though it may have been present prior to the start of the study.
- Continuous persistent disease or symptoms present at study start that worsen following the start of the study (i.e., signing of informed consent).
- Abnormal assessments, e.g., change on physical examination, ECG findings, if they represent a clinically significant finding that was not present at study start or worsened during the course of the study.

- Laboratory test abnormalities are considered as AEs if they represent a clinically significant finding, symptomatic or not, which was not present at study start or worsened during the course of the study or led to dose reduction, interruption or permanent discontinuation of study treatment. An AE is any untoward medical occurrence in a patient administered a pharmaceutical product; the event does not necessarily have a causal relationship with the treatment or usage.

Intensity of adverse events

The intensity of clinical AEs is graded on a three-point scale – mild, moderate, severe – and is reported on specific AE pages of the CRF.

If the intensity of an AE worsens during study treatment administration, only the worst intensity should be reported on the AE page. If the AE lessens in intensity, no change in the severity is required.

If the intensity of an AE with an onset date between informed consent signature and start of study treatment and which is ongoing at the start of treatment worsens after the start of study treatment, a new AE page must be completed. The onset date of this new AE corresponds to the date of worsening in intensity.

The three categories of intensity are defined as follows:

- Mild: The event may be noticeable to the patient. It does not influence daily activities, and usually does not require intervention.
- Moderate: The event may make the patient uncomfortable. Performance of daily activities may be influenced, and intervention may be needed.
- Severe: The event may cause noticeable discomfort, and usually interferes with daily activities. The patient may not be able to continue in the study, and treatment or intervention is usually needed.

A mild, moderate, or severe AE may or may not be serious, see Section 5.5.4.2. These terms are used to describe the intensity of a specific event. Medical judgment should be used on a case-by- case basis.

Seriousness, rather than severity assessment, determines the regulatory reporting obligations.

Relationship to study treatment

Each AE must be assessed by the investigator as to whether or not there is a reasonable possibility of causal relationship to the study treatment and reported as either related or unrelated. The determination of the likelihood that the study treatment caused the AE will be provided by an investigator who is a qualified physician.

4.5.4.2 Serious adverse events

Definitions of serious adverse events

An SAE is defined by the International Conference on Harmonisation (ICH) guidelines as any AE fulfilling at least one of the following criteria:

- Fatal.
- Life-threatening: refers to an event in which the patient was at risk of death at the time of the event. It does not refer to an event that hypothetically might have caused death had it been more severe.
- Requiring inpatient hospitalization, or prolongation of existing hospitalization.
- Resulting in persistent or significant disability or incapacity.
- Congenital anomaly or birth defect.
- Medically significant: refers to important medical events that may not immediately result in death, be life-threatening, or require hospitalization but may be considered to be SAEs when, based upon appropriate medical judgment, they may jeopardize the patient, and may require medical or surgical intervention to prevent one of the outcomes listed in the definitions above.

The following reasons for hospitalization are exempted from being reported:

- Hospitalization for cosmetic elective surgery, or social and/or convenience reasons.
- Hospitalization for pre-planned (i.e., planned prior to signing informed consent) surgery or standard monitoring of a pre-existing disease or medical condition that did not worsen, e.g., hospitalization for coronary angiography in a patient with stable angina pectoris.

However, complications that occur during hospitalization are AEs or SAEs (for example if a complication prolongs hospitalization).

4.5.4.3 *Reporting requirement of adverse events*

Reporting of adverse events

Irrespective of seriousness, the following adverse events are considered as adverse events of special interest and must be reported on AE page of the CRF.

In all patients:

- AEs leading to discontinuation study treatment
- Anaphylactic/anaphylactoid reactions

In patients treated with anakinra, the following AEs will be considered of special interest:

- Severe neutropenia defined as neutrophil count < 500/mm3. No other AEs are required to be reported on the AE page of the CRF

Reporting of serious adverse events

All fatal and life-threatening SAEs occurring after study drug initiation and up to the end of study visit must be reported on AE pages in the CRF and on an SAE form, regardless of the investigator-attributed causal relationship with study treatment.

No other SAEs are required to be reported on AE page of the CRF and on the SAE form to Sobi drug safety department.

Abnormal laboratory findings

Abnormal laboratory findings, if serious (fatal or life-threatening), must be reported on specific AE pages of the CRF.

Following laboratory variables are collected for the assessment of efficacy (see Section 6.5.3.2) at screening and during the treatment period, and are not required to be reported as SAEs as described above: WBC including differential counts, RBC, Hgb, platelet count, fibrinogen, PT-INR, and cardiac troponin; ALT, AST, total bilirubin, creatinine, ferritin, LDH, D-dimer, and CRP.

Follow-up of serious adverse events

Serious adverse events still ongoing at the end of study visit must be followed up until resolution or stabilization, or until the event outcome is provided, e.g., death.

Reporting procedures

All fatal and life-threatening SAEs must be reported by the investigator to the Sobi drug safety department within 24 hours of the investigator's first knowledge of the event.

All fatal and life-threating SAEs must be recorded on an SAE form, irrespective of the study treatment received by the patient, and whether or not this event is considered by the investigator to be related to study treatment.

The SAE forms must be e-mailed to the Sobi drug safety department: drugsafety@sobi.com. In copy: farmacovigilancia@sobi.com.

The investigator must complete the SAE form in English and must assess the causal relationship of the event to study treatment.

Follow-up information about a previously reported SAE must also be reported within 24 hours of receiving it. The Sobi drug safety department may contact the investigator to obtain further information.

If the patient is hospitalized in a hospital other than the study site, it is the investigator's responsibility to contact this hospital to obtain all SAE relevant information and documentation.

The reference safety document to assess expectedness of a suspected serious adverse reaction and for reporting by the Sponsor to Health Authorities, IRBs/IECs, and investigators is: Section 4.5.4.3.

4.5.4.4 Pregnancy

Reporting of pregnancy

Irrespective of the treatment received by the patient, any pregnancy occurring after study drug initiation up to the end of study visit must be reported within 24 hours of the investigator's knowledge of the event.

Pregnancies must be reported on the pregnancy form which is e-mailed to the Sobi drug safety department (drugsafety@sobi.com and farmacovigilancia@sobi.com, in copy), and on an AE page in the CRF.

Follow-up of pregnancy

Any pregnancy must be followed to its conclusion and its outcome must be reported to the Sobi drug safety department. This information will be only entered in the drug safety database.

Any AE associated with the pregnancy occurring during the study must be reported on separate AE pages in the CRF. Any SAE occurring during the pregnancy must be reported on an SAE form as described in Section 4.5.4.3.

4.5.4.5 Study safety monitoring

Clinical study safety information is monitored and reviewed on a continuous basis by the Sobi Pharmacovigilance Team (in charge of ensuring patients' safety as well as data quality).

4.5.4.6 Laboratory safety assessments

For details on laboratory assessments, see Table 3.

4.5.4.7 Vital signs

Vital signs (body temperature, blood pressure, heart rate. respiratory rate and oxygen saturation) will be measured at screening, and Days 1, 4, 7 and 15, and recorded in the CRF.

For AE reporting of abnormalities, see Section 4.5.4.3.

4.5.4.8 Electrocardiograms

A 12-lead ECG recording will be performed at screening and at Day 15 and recorded in the CRF. The ECG assessments will be performed at site and will be reported as "normal" or "abnormal". Any abnormalities should be specified. Abnormalities reported at screening should be recorded as medical history.

For AE reporting of abnormalities, see Section 4.5.4.3.

4.5.4.9 Pregnancy Test

Pregnancy is not recommended during the study. Sexual abstinence and the use of contraceptives will be recommended.

Female of childbearing potential will have a urine pregnancy test taken at screening and at Day 15. The outcome of the test will be reported as "positive" or "negative" in the CRF.

4.5.4.10 Data Review Committee

The Data Review Committee composed of experts in internal medicine will be involved in study oversight and interpretation of the study results.

4.5.4.11 Safety Review Committee

Clinical safety information (AEs, SAEs, laboratory values, ECGs, vital signs, and study-specific labs/examinations, as required) will be monitored and reviewed on a continuous basis by the Clinical Review Committee (CRC) by periodically monitoring clinical studies activities from protocol conception to database closure. There will be separate written operating procedures for the CRC.

4.5.5 Exploratory assessments

Blood samples for determination of IL-6 and selected exploratory parameters will be collected at Day 1, 7 and 15, whenever possible. The date and time of sampling will be recorded in the CRF.

All samples will be analyzed at the respective hospital local laboratory according to their standard routines.

5 Quality control and quality assurance

This study will be conducted in compliance with this protocol, and applicable regulatory requirements.

The Sponsor will establish a systematic, prioritized, risk-based approach to monitoring and, considering the current situation, will need to utilize remote monitoring. The Sponsor will develop a risk management plan with the aim to limit the contacts with patients and site personnel during the emergency period, while ensuring the patients safety and integrity, compliance with the protocol, study specific procedures and applicable regulatory requirements.

To ensure ongoing patient safety and well-being, and control risks to study critical processes, such as review of all informed consent forms and all primary efficacy variables, alternative mechanisms of oversight will be introduced (such as phone calls, video calls etc).

All information must be transcribed from clinical history to the study CRF. In this study a paper CRF will be used to capture all study data.

The study site may be subject to a quality assurance audit by the Sponsor or its representatives, as well as inspection by appropriate regulatory agencies.

It is important that the investigator(s) and the(ir) relevant personnel are available during the monitoring visits and possible audits and that sufficient time is devoted to the process.

6 Statistical plan

6.1 Determination of sample size

The study will enroll a total of 180 patients, 90 per arm according to the 1:1 randomization. The sample size has been estimated based on the following assumptions:

- The sample size is calculated in based of the data collected in the database of COVID-19 of the Complejo Hospitalario de Navarra , which currently has a total of 188 patients. After the analysis of the data, it is estimated that 15% of severe pneumonia COVID patients have required mechanical ventilation.
- For and Type I error of 0.05 and a power of 75% for each comparison under the assumption that the true success rates are 88% in the SoC group increasing to 92% in the anakinra group.
- It is needed 180 patients (90 per arm) considering a loss of 10%.
- The study will consist of two stages, with equal numbers of patients randomised into Stage 1 (treatment) and into Stage 2 (follow-up) per treatment arm.

The calculations on the operating characteristics of this design have been undertaken using the website of Sample size calculator of *National Center for Advancing Translational Sciences, National Institutes of Health, through UCSF-CTSI , Software utilities developed by Michael.Kohn@ucsf.edu, last up to date version on April 14, 2020.*

The value for the type I error has been chosen in recognition of the urgent unmet medical need to allow the identification of a signal, at least, from a statistical perspective. Having seen a statistical signal, it is then a matter of evaluating whether the observed treatment differences represent clinically relevant effects that can satisfy that unmet need.

In case indications of efficacy requires to be confirmed, additional patients may be added into this study.

In case the outcome is statistically convincing, efficacy will be considered confirmed and the results will, where warranted, be used to seek regulatory approvals.

6.2 Definition of study populations

All efficacy and safety analyses will be conducted on the all Treated population which will comprise all randomized patients receiving study treatment.

Patients will be included in the groups to which they were randomized for all evaluations of efficacy and in the groups according to treatment received for all evaluations of safety.

6.3 Overall statistical and analytical plan

Statistical analysis will be performed using PASW 21.0 statistical package.

6.3.1 General statistical issues

This design has acceptable properties in terms of the false positive potential, controlling the overall type I error at 5%, for each of the two treatment comparisons.

Secondary endpoints will be evaluated in a descriptive way. In some cases, p-value comparisons will be undertaken, but these are to be interpreted in an exploratory way.

6.3.2 Demographics and baseline characteristics

Demographic and baseline characteristics will be summarized in appropriate tabular presentations.

For measurements of continuous endpoints, summary statistics will include n, mean, median, standard deviation, minimum and maximum values. For categorical variables, summary tabulations of the number and percentage within each category (with a category for missing data) of the parameter will be presented.

6.3.3 Analysis related to primary objective

6.3.3.1 Primary endpoint

<u>Treatment success</u>

The main outcome variable is the percentage of patients requiring mechanical ventilation at 15 days after the start of treatment. The comparison of these proportions would be done by chi-square test, with significance level 0.05 and presentation in the form of absolute risk

reduction, relative risk reduction and number needed to treat, with their respective 95% confidence intervals.

6.3.3.2 *Secondary endpoints supporting the primary objective*

Time to mechanical ventilation

Analysis of the time to mechanical ventilation from the point of randomization will be undertaken by plotting Kaplan-Meier curves for each of the 2 treatment groups and by pairwise comparisons (anakinra versus standard of care) using the log rank test. Hazard ratios will be estimated using the Cox proportional hazards model and these will be presented together with 90% two-sided confidence intervals.

Change from baseline in resting SpO2 and oxygen supplementation

Change from baseline during treatment until Day 15 with measurements performed every 3 days will be summarized using descriptive statistics for each of these parameters.

Change from baseline in PaO2/FiO2

Change from baseline (screening) during treatment until Day 15 will be summarized using descriptive statistics.

Time in ICU department and in hospital

Time in ICU and time in hospital (from the point of randomization) will be analyzed at the end of the follow-up period. This endpoint will be analyzed as for time to mechanical ventilation.

6.3.4 Analysis related to secondary objective

6.3.4.1 *Mechanical vetilation at day 28.*

The percentage of patients requiring mechanical ventilation at 28 days after the start of treatment. The comparison of these proportions would be done by chi-square test, with significance level 0.05 and presentation in the form of absolute risk reduction, relative risk reduction and number needed to treat, with their respective 95% confidence intervals.

6.3.4.2 Overall mortality

Overall mortality will be analyzed at the end of the follow-up period. This endpoint will be analyzed as for time to mechanical ventilation.

6.3.4.3 Mortality at 48h, 7 days, in ICU and in hospital

Mortality at 48h, 7 days, in ICU and in hospital will be analyzed at the end of the follow-up period. This endpoint will be analyzed as for time to mechanical ventilation.

6.3.4.4 Adverse events

Reported AEs during the study will be coded using MedDRA. The incidence of AEs will be summarized in frequency tables by treatment, system organ class, preferred term and maximum severity. Separate tabulations will be performed for serious and non-serious AEs.

6.3.5 Analysis related to exploratory objective

<u>Change from baseline in hyperinflammatory parameters</u>

Change from baseline during treatment until Day 15 with measurements performed days 1, 4, 7 and 15 of the following parameters will be summarized using descriptive statistics:

- Ferritin
- LDH
- D-dimers
- CRP

<u>Change from baseline in other relevant laboratory parameters</u>

Change from baseline during treatment until Day 15 with measurements performed days 1, 4, 7 and 15 or until hospital discharge of the following parameters will be summarized using descriptive statistics:

- WBC with differential counts
- RBC
- Hb

- Platelet count
- Fibrinogen
- Prothrombin Time
- Cardiac troponin
- Liver tests (AST, ALT, total bilirubin levels)
- Creatinine

6.3.6 Interim analysis

The study will consist of one interim analysis (Stage 1 - treatment) and a final analysis (Stage 2 – follow-up), with approximately the same numbers of patients randomized into Stage 1 and into Stage 2 per treatment arm. The Stage 1 interim analysis will be conducted after the accrual of at least 25 patients per treatment arm.

6.3.7 Multiple comparison/multiplicity

There will be no adjustments for multiplicity for the two pairwise treatment comparisons for the primary endpoint. Multiplicity for the sequential comparisons at the end of Stage 1 and at the end of Stage 2 are however accounted for by the design.

6.3.8 Handling of missing data

There will be no computation of missing data.

7 Data collection, handling and record keeping

7.1 Data standards

Collection of data should be performed in the CDASH format, according to the CDISC. The standards should be used to the extent possible and/or required for the specific study/project. The minimum requirement of the CDISC standard is to collect all core variables specified as 'Required' in the Study Data Tabulation Model format.

7.2 Case report form

A CRF is required and should be completed for each included patient. In this study, a paper CRF will be used to capture all study data except for the scheduled laboratory results from the hospital´s local laboratory.

It is the responsibility of the Principal Investigator to ensure the completeness, legibility, and accuracy of the data reported in the CRF, to review and to confirm these by proving his/her signature, thus approving all CRFs pages. The Principal Investigator must sign off each visit in the CRF using black ballpoint pen, in addition to providing a final signature of the completed CRF. These signatures serve to attest that the information contained on these CRFs is correct. At all times, the investigator has final responsibility for the accuracy and authenticity of all clinical and laboratory data entered on the CRFs.

In either case, it is important that the original CRF pages remain at site.

7.3 Source data

Patient source documents are the patient's medical records maintained at the study site. In most cases, the source documents will be the hospital's or the physician's chart, including laboratory test results, radiology results, EKG etc. In those cases, the information collected in the CRFs must match those charts.

A separate source document location agreement will be completed and signed by the Principal Investigator and the site monitor.

Source data should be attributable, legible, contemporaneous, original, accurate, and complete. Changes to source data should be traceable, should not obscure the original entry, and should be explained if necessary (e.g. via an audit trial).

7.4 Protocol deviations

A protocol deviation is generally an unplanned excursion from the protocol that is not implemented or intended as a systematic change. The investigator is responsible for ensuring the study is conducted in accordance with the procedures and evaluations described in this protocol and must protect the rights, safety, and welfare of patients. The investigator should not implement any deviation from, or changes of, the protocol, unless it is necessary to eliminate an immediate hazard to study patients.

A protocol waiver is a documented prospective approval of a request from an investigator to deviate from the protocol. Protocol waivers are strictly prohibited.

The investigator will assure that deviations are reported and documented in accordance with IEC and applicable regulatory requirements.

7.5 Database closure

Prior to database closure, all tasks or criteria defined in the data management plan must be completed and documented. The study database must be locked before generation of any results. The database lock will be approved by relevant study personnel and all edit accesses will be removed. Following database closure, the study database can only be unlocked in case critical errors, affecting the main conclusions of the study, are discovered.

7.6 Record retention

The investigator should maintain a record of the location(s) of investigator's essential documents as defined in the ICH GCP Guideline [1] including source documents and should have control of and continuous access to all essential documents and records generated by the investigator/institution before, during, and after the study.

All documents and data relating to the study will be kept securely by the investigator in a secure file and/or electronically. The storage system used during the study and for archiving

(irrespective of the type of media used) should provide for document identification, version, history, search and retrieval. The data will be available for evaluation and/or audits from Health Authorities, Sponsor or Sponsor's representatives.

When a copy is used to replace an original document (e.g. source documents, CRF), the copy should fulfill the requirements for certified copy as defined in ICH GCP Guideline [1].

The records should be retained by the Investigator as specified in the Clinical Trial Agreement and in accordance with local regulations.

If the investigator relocates, retires, or for any reason withdraws from the study, the study records may be transferred to an acceptable designee, such as another investigator or another institution.

8 End of study

The end of this study is defined as the date of the last patient's last visit/end of study call.

9 Sponsor's discontinuation criteria

The Sponsor and GEAS through the Principal Coordinating Investigator, reserve the right to discontinue the study prior to inclusion of the intended number of patients but intends only to exercise this right for valid scientific or administrative reasons. After such a decision, the investigator must contact all participating patients within 30 days. All study materials must be collected and all the CRFs completed to the greatest extent possible.

10 Dissemination and publication of results

The study information and post study results regardless of outcome will be published on a publicly accessible website in accordance with applicable laws and regulations, e.g., on www.clinicaltrials.gov and EudraCT. The results of this study will be published within 12 months of the end of study.

The Sponsor is committed to publishing study results in a complete, accurate, balanced, transparent and timely manner. The Sponsor follows the principles of the International Committee of Medical Journal Editors (ICMJE) recommendations for the conduct, reporting, editing, and publication of scholarly work in medical journals including criteria for authorship.

The data from this study will be considered for reporting at a scientific meeting or for publication in a scientific journal. The Sponsor will be responsible for these activities and will work with the investigators to determine how the publication is written, the number and order of authors, the journal or scientific meeting to which it will be submitted, and other related issues. The results of the study, or any part thereof, shall not be published without the prior written consent and approval of the Sponsor and GEAS through the Principal Coordinating Investigator, such consent and approval not to be unreasonably withheld.

11 Reference list

1. Munster VJ, Koopsman M, Van Doremalen N, Van Riel D, de Wit E. A novel coronavirus emerging in Chin-Key questions for impact assessment . N Engl J Med 2020; 382:692-694
2. Guan W-J, Ni Z-Y, Hu Y, Liang W-H, Ou C-Q, He J-X, et al. Clinical Characteristics of Coronavirus Disease 2019 in China. N Engl J Med [Internet]. 2020; Disponible en: http://www.ncbi.nlm.nih.gov/pubmed/32109013
3. Ruan Q, Yang K, Wang W, Jiang L, Song J. Predicciones clínicas de mortalidad por COVID-19 basadas en un análisis de datos de 150 pacientes de Wuhan, China. Intensive Care Med [Internet]. 2020; se puede consultar en: http://www.ncbi.nlm.nih.gov/pubmed/32125452
4. Weiss P, Murdoch DR. Curso clínico y riesgo de mortalidad de COVID-19 severo. Lancet. 2020;395(10229):1014–1015.
5. Xu Z, Shi L, Wang Y, Zhang J, Huang L, Zhang C, et al. Hallazgos patológicos de COVID-19 asociados con el síndrome de dificultad respiratoria aguda. Lancet Respir Med [Internet]. 2020;2600(20):19–21. Se puede consultar en: http://dx.doi.org/10.1016/S2213-2600(20)30076-X
6. Li G, Fan Y, Lai Y, Han T, Li Z, Zhou P, et al. Infecciones por virus de la corona y respuestas inmunológicas. 6. J Med Virol. 2020;92(4):424–32.
7. Behrens EM, Koretzky GA. Revisión: Síndrome de Tormenta de Citocina: Mirando hacia la era de la medicina de precisión. Artritis Reumatol. 2017;69(6):1135–43.
8. Sönmez HE, Demir S, Bilginer Y, Özen S. Tratamiento de Anakinra en el síndrome de activación de macrófagos: una experiencia de centro único y revisión sistémica de la literatura. Clin Rheumatol. 2018;37(12):3329–35.
9. Yoo DH. Biológicos para el tratamiento de la enfermedad de Still de inicio en la edad adulta. Expert Opinion Biol Ther [Internet]. 2019;19(11):1173–90. Se puede consultar en: https://doi.org/10.1080/14712598.2019.1652591
10. Sönmez HE, Demir S, Bilginer Y, Özen S. Tratamiento de Anakinra en el síndrome de activación de macrófagos: una experiencia de un solo centro y una revisión sistémica de la literatura. Clin Rheumatol. 2018;37(12):3329–3335.
11. Henderson LA, Cron RQ. Síndrome de activación de macrófagos y linfohistiocitosis hemofágica secundaria en los trastornos inflamatorios infantiles: Diagnóstico y tratamiento. Medicamentos pediátricos. 2020;22(1):29–44.
12. Gattinoni L, Chiumello D, Caironi P, Busana M , Romitti F et al. COVID-19 pneumonia: different respiratory treatment for different phenotypes? Intensive Care Medicine.DOI 10.1007/s00134-020-06033-2

13. Shakoory B, Carcillo JA, Chatham WW, et al. Interleukin-1 Receptor Blockade Is Associated With Reduced Mortality in Sepsis Patients With Features of Macrophage Activation Syndrome: Reanálisis de un ensayo anterior de fase III. Crit Care Med. 2016;44(2):275–281.
14. Granowitz E et al. Pharmacokinetics, safety, and immunomodulatory effects of human recombinant interleukin-1 receptor antagonist in healthy humans. CYTOKINE 1992; 4(5):353-360
15. Badheka A et al. Use of an interleukin-1 receptor antagonist for suspected sepsis with hyperinflammation in children. Crit Care Med. 2019; 48:1561
16. Opal S et al. Confirmatory interleukin-1 receptor antagonist trial in severe sepsis: A phase III, randomized, double-blind, placebo-controlled, multicenter study. Crit Care Med. 1997; 25:1115-1124
17. Fisher C et al. Initial evalutation of human recombinant interleukin-1 receptor antagonist in the treatment of sepsis syndrome: A randomized, open-label, placebo-controlled multicenter study. CRITICAL CARE MEDICINE 1994; 22(1):12-21
18. Galea J et al. Intravenous anakinra can achieve experimentally effective concentrations in the central nervous system within a therapeutic time window results of a dose-ranging study. JCBFM. 2011; 31:439-447
19. Cao B, Wang Y, Wen D, Liu W, Wang J, Fan G, et al. A Trial of Lopinavir-Ritonavir in Adults Hospitalized with Severe Covid-19. N Engl J Med [Internet]. 2020;1–13. Se puede consultar en: http://www.ncbi.nlm.nih.gov/pubmed/32187464
20. Huang C et al. Clinical features of patients infected with 2019 novel coronavirus in Wuhan. Lancet 2020; 395(10223):497-506
21. Ruan Q et al. Clinical predictors of mortality due to COVID-19 based on an analysis of data of 150 patients from Wuhan, China. Intensive Care Med. 2020; (published online Mar 03) doi: 10.1007/s00134-020-05991-x
22. Mehta P et al. COVID-19: consider cytokine storm syndromes and immunosuppression. Lancet 2020; (published online Mar 13) doi.org/10.1016/ S0140-6736(20)30628-0
23. Xu X et al. Effective Treatment of Severe COVID-19 Patients with Tocilizumab ChinaXiv:202003.00026v1
24. Locatelli F et al. Safety and Efficacy of Emapalumab in Pediatric Patients with Primary Hemophagocytic Lymphohistiocytosis. Blood 2018;132: LBA-6; doi: https://doi.org/10.1182/blood-2018-120810

25. Benedetti F et al. Emapalumab, an interferon gamma (IFN-γ)-blocking monoclonal antibody, in patients with Macrophage Activation Syndrome (MAS) complicating systemic Juvenile Idiopathic Arthritis (sJIA). Annals of Rheumatic Diseases, 2019; 78 (Suppl 2):178.1-178
26. Eloseily EM et al. Benefit of anakinra in treating pediatric secondary hemophagocytic lymphohistiocytosis. Arthritis Rheumatol. 2020;72:326-334 doi: 10.1002/art.41103
27. Adam Monteagudo L et al. Continuous Intravenous Anakinra Infusion to Calm the Cytokine Storm in Macrophage Activation Syndrome. ACR Open Rheumatol. 2020 Apr 8. doi: 10.1002/acr2.11135. [Epub ahead of print]

12 Anexo

Participating centers and investigators

CENTERS	INVESTIGATOR
Complexo Hospitalario Universitario de Santiago	Maria Carmen Freire Dapena
Hospital Universitario Son Espases	Lucio Pallarés Ferreres
Complejo Hospitalario de Navarra, Pamplona	Patricia Fanlo Mateo
Hospital Universitario y Politécnico La Fe, Valencia	Jose Antonio Todolí Parra
Hospital Universitario Vall d'Hebron, Barcelona	Segundo Buján Rivas
Hospital Universitario La Paz, Madrid	Angel Robles- Marhuenda
	Francisco Arnalich Fernández
Hopital Clínic, Barcelona	Sergio Prieto González
Hospital Clínico Lozano Blesa, Zaragoza	Borja del Carmelo GraciaTello
Hospital Ramón y Cajal, Madrid	Andrés González García
Hospital Álvaro Cunqueiro, Vigo	Ana Argibay Filgueira
Hospital Universitario Miguel Servet, Zaragoza	Mercedes Pérez Conesa
