# Supplementary material for: Efficacy and Safety of Anakinra Plus Standard of Care for Patients With Severe COVID-19: A Randomized Phase 2/3 Clinical Trial
Source: JAMA Netw Open. 2023 Apr 7;6(4):e237243. doi: 10.1001/jamanetworkopen.2023.7243 (PMC10082404; doi:10.1001/jamanetworkopen.2023.7243)
Supplement: Supplement 2. — eAppendix 1. List of Participating Centers eAppendix 2. Supplemental Methods eTable 1. Baseline Vital Signs, Respiratory Function, and Laboratory Values eTable 2. Anakinra Administration and Discontinuation eTable 3. Methylprednisolone Pulse Therapy Administered Until Day 15 eTable 4. Treatments for COVID-19 Received Before and After Randomisation During the Trial eTable 5. Dose and Duration of Treatments for COVID-19 Received Before and After Randomisation During the Trial eTable 6. Sensibility Analysis Results for the Primary Outcome for Different Scenarios Regarding Assumptions of the Lost to Follow-up Patients eTable 7. Vital Signs and Respiratory Function by Timepoint eTable 8. Levels of Hyperinflammatory Parameters and Other Relevant Laboratory Parameters by Timepoint eTable 9. Evolution of Hyperinflammatory Parameters Over Time eTable 10. Primary and Secondary Efficacy Outcomes in Patients With SCOPE Score ≥ 6 eTable 11. Adjusted Multivariate Cox Regression Hazard Model to Estimate Risk Factors for Mechanical Ventilation eTable 12. Cox Regression Hazard Model to Estimate Risk Factors for Survival eTable 13. Logistic Regression Analysis Model of Factors Affecting Viral Clearance eTable 14. Rescue Therapy eTable 15. Imaging Assessment eTable 16. Symptoms of COVID-19 at Day 28 eTable 17. Serious Adverse Events eFigure 1. Evolution of the First Panel of Exploratory Outcomes eFigure 2. Evolution of the Second Panel of Exploratory Outcomes eReferences [file jamanetwopen-e237243-s002.pdf]

Supplemental Online Content

Fanlo P, Gracia-Tello BC, Fonseca Aizpuru E, et al; GEAS-SEMI Group. Efficacy and safety of anakinra plus standard of care for patients with severe COVID-19: a randomized phase 2/3 clinical trial. *JAMA Netw Open*. 2023;6(4):e237243. doi:10.1001/jamanetworkopen.2023.7243

eAppendix 1. List of Participating Centers

eAppendix 2. Supplemental Methods

eTable 1. Baseline Vital Signs, Respiratory Function, and Laboratory Values

eTable 2. Anakinra Administration and Discontinuation

eTable 3. Methylprednisolone Pulse Therapy Administered Until Day 15

eTable 4. Treatments for COVID-19 Received Before and After Randomisation During the Trial

eTable 5. Dose and Duration of Treatments for COVID-19 Received Before and After Randomisation During the Trial

eTable 6. Sensibility Analysis Results for the Primary Outcome for Different Scenarios Regarding Assumptions of the Lost to Follow-up Patients

eTable 7. Vital Signs and Respiratory Function by Timepoint

eTable 8. Levels of Hyperinflammatory Parameters and Other Relevant Laboratory Parameters by Timepoint

eTable 9. Evolution of Hyperinflammatory Parameters Over Time

eTable 10. Primary and Secondary Efficacy Outcomes in Patients With SCOPE Score ≥ 6

eTable 11. Adjusted Multivariate Cox Regression Hazard Model to Estimate Risk Factors for Mechanical Ventilation

eTable 12. Cox Regression Hazard Model to Estimate Risk Factors for Survival

eTable 13. Logistic Regression Analysis Model of Factors Affecting Viral Clearance

eTable 14. Rescue Therapy

eTable 15. Imaging Assessment

eTable 16. Symptoms of COVID-19 at Day 28

eTable 17. Serious Adverse Events

eFigure 1. Evolution of the First Panel of Exploratory Outcomes

eFigure 2. Evolution of the Second Panel of Exploratory Outcomes

eReferences

This supplemental material has been provided by the authors to give readers additional information about their work.

© 2023 Fanlo P et al. *JAMA Network Open.*

eAppendix 1. List of Participating Centers

Complejo Hospitalario de Navarra, Pamplona (Spain)

Hospital Lozano Blesa, Zaragoza (Spain)

Hospital Son Espases, Palma de Mallorca (Spain)

Hospital Clinic, Barcelona (Spain)

Hospital Vall d´Hebrón, Barcelona (Spain)

Hospital La Fe, Valencia (Spain)

Hospital La Paz, Madrid (Spain)

Hospital Ramón y Cajal, Madrid (Spain)

Complejo Hospitalario de Vigo, Vigo (Spain)

Complejo Hospitalario Universitario de Santiago, Santiago de Compostela (Spain)

Hospital Miguel Servet, Zaragoza (Spain)

Hospital Cabueñes, Gijón (Spain)

© 2023 Fanlo P et al. *JAMA Network Open.*

eAppendix 2. Supplemental Methods

Inclusion and exclusion criteria

Inclusion criteria: A patient must fulfill the following criteria in order to be included in the study:

- Age 18-80 years.
- Severe pneumonia COVID-19 defined as:
  - Nasopharyngeal smear with RCP positive for SARS-CoV-2
  - X-Rays (or other technique) pulmonary infiltrates compatible with pneumonia.
  - 1 or more of the following criteria:
    - Ambient air oxygen saturation <= 94% measured with a pulse oximeter.
    - Pa:FiO2 (partial pressure O2/fraction of inspired O2) <=300.
    - Sa:FiO2 (O2 saturation measured with pulse oximeter/ fraction of inspired O2) <=350.
- High suspicion of CSS that could resemble MAS-like: represented by IL-6 values > 40 pg/mL and/or ferritin >500 ug/L and/or PCR > 30 mg/L (rationale: ≥ 5 upper normal limit) and/or LDH >300 UI/L. We have chosen these parameters because they are implemented in all the participating hospitals, they are a reflection of the cytokine storm and they have also been significant in terms of predicting mortality in patients with COVID-19 (9).
- Written informed consent. The protocol will be explained to the patient in front of a nurse who will act as a legal witness by signing the document on behalf of the patient.

Exclusion criteria: The presence of any of the following will exclude a patient from inclusion in the study:

- Need for oro-tracheal intubation and/or invasive mechanical ventilation at the start of the study.
- AST/ALT with values greater than 5 times normal levels.
- Neutrophils < 1.500 cell/mmc.
- Platelets < 50.000 cell/mmc.
- Sepsis or pneumonia documented by other pathogens than SARS-CoV-2.
- Existence of any life-threatening comorbidity or any other medical condition that, in the investigator's opinion, makes the patient unsuitable for inclusion.
- Inability to obtain informed consent.
- Positivity for HBV or HCV.
- Patient with active tuberculosis (It is at the discretion of the researcher to perform the tuberculin test on the patient in the screening period if it is considered a risk population and on patients with active tuberculosis manifestations).
- Pregnancy.
- Use of other previous or concomitant biological treatments. Patients in concomitant treatment with other biologicals that may interfere will be excluded: tocilizumab, canakinumab, TNFalfa inhibitors, JAKiinibs
- Severe renal dysfunction (estimated glomerular filtration rate ≤ 30 ml / min / 1.73

© 2023 Fanlo P et al. *JAMA Network Open.*

m2) or receive continuous renal replacement therapy, hemodialysis or peritoneal dialysis.

- Uncontrolled hypertension (sitting systolic blood pressure > 180 mmHg or diastolic blood pressure > 110 mmHg).
- Administration of plasma from convalescent patients who have recovered from

  SARS-CoV-2 infection.

- History of hypersensitivity or allergy to any component of the study drug.
- Enrollment in another concurrent intervention clinical trial, or intake of an investigational medication within three months or 5 half-lives prior to inclusion in this study, if deemed to interfere with the objectives of this study as assessed by the investigator.
- Predictable inability to cooperate with given instructions or study procedures.

Procedures

Anakinra Dose selection: Anakinra was given intravenously 100 mg four times (every 6 hours) a day (total daily dose 400 mg) for a maximum of 15 days if needed according to clinical response. The dose was chosen based on the range of doses used in clinical trials of anakinra in critically ill patients. More precisely, anakinra is approved for the chronic treatment of a number of inflammatory diseases as a subcutaneous treatment (at doses of 100 mg/day or in weight-based doses of up to 8 mg/kg/day). The i.v. administration of anakinra has been studied in clinical trials in healthy volunteers and in critically ill patients with sepsis and hyper-inflammation at variable i.v. doses up to 3500 mg/day over 72 hours: e.g., 2 mg/kg/hour, 20 mg/kg/day (<40 kg) and 916 mg/day (> 40kg), bolus of 100 mg followed by infusion of 2 mg/kg/hour. No safety concerns emerged in these studies.[1,5] A study in children with systemic onset juvenile arthritis complicated by refractory macrophage activation syndrome was currently ongoing at study design protocol (NCT02780583) in which anakinra was administered at dose of 10 mg/kg/day to a maximum of dose of 200 mg/day divided every 12 hours (for children ≤40 kg) or 5 mg/kg/day up to a maximum dose of 400 mg/day divided every 6 hours (children > 40 kg and adults). Based on the above, in this study anakinra was administered at a total dose of 400 mg per day, divided in 4 i.v. doses of 100 mg every 6 hours.

Standard of care-doses: The standard of care included hydroxychloroquine (400 mg two times daily, every 12 hours for 5 days) and/or lopinavir-ritonavir (800/200 mg two times daily, every 12 hours for 5-7 days) and/or azithromycin (500 mg once daily for 5 days). Background treatment with intravenous methylprednisolone pulse therapy (250-500 mg daily) administered for three consecutive days at any time during the study was recommended based on its use with anakinra in secondary hemophagocytic lymphohistiocytosis and MAS.

Concomitant therapy: The concomitant use of other IL-1 inhibitors, IL-6 inhibitors, TNF inhibitors and JAK inhibitors was not allowed. Antimicrobial therapy and prophylaxis are not limited. Permitted concomitant medications included analgesics, antibiotics, antifungals and antiviral drugs. In case of disease worsening in terms of worsening of respiratory status, the patients could be withdrawn from the study and receive rescue medication if deemed necessary by the attending physician.

Rescue Medication: All patients must have access to rescue treatment with independent of the assigned arm. If a patient withdraws due to worsening of disease, patient should be treated as a non- responder. In the case of a patient starts to worsening independent of the assigned arm, the patient will be able to receive treatments that are protocolized at their hospital. The patient could be treated with pulses of steroids, intravenous inmunoglobulins, betainterferon , immunosuppressants agents such as cyclosporin, tacrolimus and other biologic agents such as baricitinib, tocilizumab, sarilumab and ruxolitinib. If the patient need therapy with biological drugs, they should be removed from the study.

© 2023 Fanlo P et al. *JAMA Network Open.*

Trial conduct:

Clinical data were recorded using an eCRF. To ensure data quality, remote data monitoring was performed by dedicated trial staff who were independent of the site investigators. The trial was monitored by a contract research organization (Dynamic Science, Evidenze Clinical Research).

Imaging findings, including chest X-ray and computed tomography were interpreted by radiologists from each participant site that were entirely independent and unaware whether the patients were included in a clinical trial.

© 2023 Fanlo P et al. *JAMA Network Open.*

eTable 1. Baseline Vital Signs, Respiratory Function, and Laboratory Values

Variable	Anakinra group	Standard of care group
Systolic blood pressure, mmHg	124·0 (114·5-131·0; n=89)	125·0 (113·0-134·0; n=89)
Diastolic blood pressure, mmHg	74·0 (66·5-83·0; n=89)	76·0 (68·0-82·0; n=89)
Heart rate, bpm	76·5 (69·0-86·0; n=88))	78·0 (70·0-89·0; n=87))
Respiratory rate, bpm	20·0 (18·0-24·0; n=76)	20·0 (16·3-23·5; n=76)
PaO_2, mmHg	62·6 (56·0-69·8; n=56)	63·5 (57·0-74·5; n=56)
FiO_2, mmHg	24·0 (21·0-32·0)	24·0 (21·0-30·8)
SpO_2:FiO_2	380·0 (280·0-440·0; n=86)	380·0 (300·0-440·0; n=75)
Resting SpO_2, %		
Measurement 1	94·0 (92·0-97·0; n=87)	94·0 (92·0-95·0; n=81)
Measurement 2	94·0 (93·0-96·0; n=77)	95·0 (93·0-96·0; n=68)
Measurement 3	94·0 (92·0-96·0; n=75)	93·0 (93·0-96·0; n=67)
Other relevant laboratory parameters		
Platelet count, $\times 10^9$/L	225·5 (177·8-302·5; n=88)	239·5 (183·8-325·5; n=86)
Haemoglobin	14·1 (13·1-15·2; n=88)	13·8 (12·6-14·8; n=86)
Red blood cells, $\times 10^{12}$/L	4·7 (4·4-5·1; n=88)	4·7 (4·3-5·0; n=86)
Erythrocyte sedimentation rate, mmh	47·0 (30·0-63·0; n=21)	50·0 (29·0-88·0; n=21)
Fibrinogen, mg/dL	713·0 (621·5-785·8; n=76)	714·0 (609·5-819·8; n=80)
Prothrombin time, s	10·9 (1·1-12·6; n=82)	1·3 (1·1-12·6; n=84)
Cardiac troponin, ng/mL	0·0 (0·0-5·0; n=55)	0·0 (0·0-6·9; n=58)
AST, U/L	36·5 (25·8-50·0; n=82)	39·0 (26·5-56·5; n=85)
ALT, U/L	35·0 (24·0-61·0; n=87)	38·5 (25·0-61·8; n=84)
Total bilirubin, mg/L	0·5 (0·4-0·6; n=67)	0·5 (0·4-0·6; n=71)
Creatinine, mg/L	0·8 (0·7-1·0; n=88)	0·8 (0·7-0·9; n=71)
Disease severity		
11-point WHO-CPS score*	5·0 (5·0-5·0; n=89)	5·0 (5·0-5·0; n=87)
4	1 (1·1%)	13 (14·9%)
5	73 (82·0%)	54 (62·1%)
6	15 (16·9%)	20 (23·0%)
SCOPE score		
<6	13 (17·6%; n=74)	9 (13·4%; n=67)
≥6	61 (82·4%; n=74)	58 (86·6%; n=67)

Data are mean (SD), n (%), or median (IQR). Percentages might not total 100% due to rounding. AST: aspartate aminotransferase. ALT: alanine transaminase. FiO2: fractional of inspired oxygen. HRTC: high resolution computed tomography. PaO2: arterial partial pressure of oxygen. SCOPE: Severe COvid Prediction Estimate score. WHO-CPS: World Health Organization clinical progression scale. * Comparison of the distribution of the scores of the 11-point WHO-CPS at screenig was significantly different between groups (p=0·0001).

© 2023 Fanlo P et al. *JAMA Network Open.*

eTable 2. Anakinra Administration and Discontinuation

Variable	Anakinra plus standard of care (n=89)
Total doses received	60·0 (47·0-60·0; n=89)
Doses received	
<30 doses	14 (15·7%)
≥30 doses	24 (27·0%)
60 doses	50 (56·2%)
61 doses	1 (1·1%)
Daily dose, mg	375 (367·-375·0; n=89)
Duration of treatment, days	16·0 (12·5-16·0, n=89)
Cumulative dose	
Treatment interruption	4 (4·5%, n=89)*
Treatment discontinuation, n (%)	27 (30·3%, n=89)
Adverse event	15 (55·6%, n=27)
Clinical improvement	3 (27·3%, n=27)
Prohibited medication	2 (7·4%, n=27)
Patient decision	3 (27·3%, n=27)†
Informed consent withdrawal	1 (3·7%, n=27)
Withdrawal by the investigator	2 (7·4%, n=27)‡
Mistake	1 (3·7%, n=27)

Data are n (%) or median (IQR). *One treatment interruption; †Patient decides to discontinue study treatment but agrees to continue participating in the study; ‡The ICU attending physicians decided to discontinue anakinra

eTable 3. Methylprednisolone Pulse Therapy Administered Until Day 15

	Anakinra group (n=89)	Standard of care group (n=87)	p-value
At least one dose	33 (37·1%)	34 (39·1%)	0·88*
Number of doses received	6·0 (3·0-13·0)	8·5 (3·0-12·5)	0·13†
Duration, days	3·0 (3·0-10·0)	6·5 (3·0-11·0)	0·19†
Daily dose	91·0 (40·0-250·0)	79·9 (39·3-184·0)	0·56†

Data are n (%) or median (IQR). *Chi-square; †Mann-Whitney

© 2023 Fanlo P et al. *JAMA Network Open.*

eTable 4. Treatments for COVID-19 Received Before and After Randomisation During the Trial

	Anakinra plus standard of care (n=89)			Standard of care (n=87)		
	**Before randomisation**	**After randomisation (concomitant)**	**Any time before or after randomisation**	**Before randomisation**	**After randomisation**	**Any time before or after randomisation**
Corticosteroids						
Dexamethasone	0 (0·0%)	9 (10·1%)	54 (60·7%)	0 (0·0%)	6 (6·9%)	49 (56·3%)
Methylprednisolone	0 (0·0%)	3 (3·4%)	9 (10·1%)	1 (1·1%)	3 (3·4%)	11 (12·6%)
Prednisone	0 (0·0%)	7 (7·9%)	7 (7·9%)	0 (0·0%)	7 (8·1%)	7 (8·0%)
Antiviral drugs						
Remdesivir	0 (0·0%)	4 (4·5%)	22 (24·7%)	1 (1·1%)	0 (0·0%)	14 (16·1%)
Lopinavir-ritonavir	1 (1·1%)	0 (0·0%)	4 (4·5%)	1 (1·1%)	0 (0·0%)	4 (4·6%)
Azithromycin	0 (0·0%)	1 (1·1%)	8 (9·0%)	0 (0·0%)	0 (0·0%)	13 (14·9%)
Hydroxychloroquine	1 (1·1%)	1 (1·1%)	8 (9·0%)	1 (1·1%)	0 (0·0%)	5 (5·7%)

Data are n (%)

© 2023 Fanlo P et al. *JAMA Network Open.*

eTable 5. Dose and Duration of Treatments for COVID-19 Received Before and After Randomisation During the Trial

	Anakinra group (n=89)			Standard of care group (n=87)			Differences between groups		
	**At least one dose**	**Duration of treatment**, days	**Daily dose**	**At least one dose**	**Duration of treatment**, days	**Daily dose**	**Proportion of patients**	**Duration of treatment**	**Daily dose**
Corticosteroids									
Dexamethasone	54 (60·7; n=89)	9·5 (7·0-12·0; n=54)	6·0 (6·0-9·0; n=54)	49 (56·3; n=87)	8·0 (5·0-10·0; n=49)	6·0 (6·0-8·0; n=49)	0·65*	0·03†	0·63†
Prednisone	7 (7·9; n=89)	9·0 (5·0-11·0; n=7)		7 (8·0; n=87)	3·0 (1·0-4·0; n=7)		1·00	0·02†	
Antiviral drugs									
Remdesivir	22 (24·7; n=89)	5·5 (5·0-6·0; n=22)	100·0 (100·0-125·0; n=22)	14 (16·1; n=87)	5·0 (4·0-6·0; n=14)	135·0 (100·0-200·0; n=14)	0·19*	0·38†	0·14†
Lopinavir-ritonavir	4 (4·5; n=89)	5·5 (2·0-6·0; n=4)	800·0 (500·0-800·0; n=4)	4 (4·6; n=87)	6·5 (5·0-9·5; n=4)	800·0 (800·0-1400·0; n=4)	1·00	0·19‡	0·34†
Azithromycin	8 (9·0; n=89)	6·0 (4·5-7·0; n=8)	500·0 (500·0-500·0; n=8)	13 (14·9; n=87)	6·0 (4·5-6·0; n=13)	500·0 (500·0-500·0; n=13)		0·59	..
Hydroxychloroquine	8 (9·0; n=89)	5·0 (2·9-5·0; n=8)	506·7 (400·0-740·0; n=8)	5 (5·7; n=87)	4·0 (3·0-5·0; n=5)	560·0 (400·0-700·0; n=5)	0·57*	0·44*	0·83*

Data are n (%) or median (IQR). *Chi-square; †Mann-Whitney; ‡T-test

© 2023 Fanlo P et al. *JAMA Network Open.*

eTable 6: Sensibility Analysis Results for the Primary Outcome for Different Scenarios Regarding Assumptions of the Lost to Follow-up Patients

	Mechanical ventilation	Not mechanical ventilation	Total	RR (for not requiring ventilation) (95%CI) (p-value)
**Scenario conducted ITT (with 9 patients lost to follow up as 'missing')**				
SoC	11	67	78	0.90 (0.77, 1.04) (p=0.16)
Anakinra	19	64	83	
**Scenario assuming 9 missing as 'not requiring mechanical ventilation' or 'best case'**				
SoC	11	67+6 = 73	84	
Anakinra	19	64+3 = 67	86	0.90 (0.78, 1.03) (p=0.13)
**Scenario assuming 9 missing as 'requiring mechanical ventilation' or 'worst case'**				
SoC	11+6=17	67	84	
Anakinra	19+3=22	64	86	0.93 (0.79, 1.10) (p=0.42)

© 2023 Fanlo P et al. *JAMA Network Open.*

eTable 7. Vital Signs and Respiratory Function by Timepoint

Variable		Anakinra group	Standard of care group	p-value
Temperature, ºC	**Baseline**	36·2 (36·0-36·7; n=89)	36·2 (35·7-36·4; n=87)	0·09
	**Day 4**	36·1 (35·7-36·5; n=85)	36·2 (35·8-36·4; n=79)	0·53
	**Day 7**	36·0 (35·7-36·2; n=50)	36·1 (35·8-36·2; n=63)	0·54
	**Day 15**	36·1 (35·7-36·5; n=50)	36·2 (35·8-36·5; n=48)	0·30
Systolic blood pressure	**Baseline**	124·0 (114·5-131·0)	125·0 (113·0-134·0)	0·75
	**Day 4**	127·0 (117·5-136·0; n=85)	123·0 (112·0-136·0; n=79)	0·08
	**Day 7**	125·0 (116·8-138·3; n=74)	121·0 (110·0-133·0; n=63)	0·03
	**Day 15**	126·5 (113·5-139·0; n=52)	122·0 (111·0-130·0; n=45)	0·05
Diastolic blood pressure	**Baseline**	74·0 (66·5-83·0)	76·0 (68·0-82·0)	0·74
	**Day 4**	76·0 (71·0-81·5; n=85)	73·0 (63·8-80·0; n=78)	0·01
	**Day 7**	75·0 (67·0-82·3; n=74)	73·0 (68·0-83·0; n=63)	0·94
	**Day 15**	73·0 (65·8-80·8; n=52)	70·0 (62·0-78·5; n=45)	0·29
Heart rate	**Baseline**	76·5 (69·0-86·0; n=88)	78·0 (70·0-89·0; n=87)	0·78
	**Day 4**	73·5 (63·3-81·0; n=84)	74·0 (64·0-81·0; n=79)	0·75
	**Day 7**	73·5 (60·8-81·0; n=74)	76·0 (67·8-84·0; n=62)	0·03
	**Day 15**	78·0 (70·0-88·0; n=52)	78·0 (71·0-90·0; n=47)	0·83
Respiratory rate	**Baseline**	20·0 (18·0-24·0; n=76)	20·0 (16·3-23·5; n=76)	0·55
	**Day 4**	18·0 (15·0-21·0; n=51)	18·0 (16·0-20·0; n=54)	0·53
	**Day 7**	17·0 (14·0-20·0; n=45)	19·0 (15·0-22·0; n=39)	0·05
	**Day 15**	14·5 (12·0-20·0; n=36)	18·0 (13·5-21·0; n=41)	0·11
Oxygen saturation on air at rest, %	**Baseline**	93·0 (90·0-93·5; n=37)	93·0 (90·0-94·0; n=38)	0·68
	**Day 4**	95·5 (94·0-97·0; n=32)	96·0 (94·0-97·0; n=43)	0·68
	**Day 7**	96·0 (94·0-97·0; n=38)	96·0 (94·0-98·0; n=38)	0·66
	**Day 15**	97·0 (95·0-98·0; n=45)	96·5 (96·0-98·0; n=44)	0·79
PaO_2	**Baseline**	62·6 (56·0-69·8; n=56)	63·5 (57·0-74·5; n=56)	0·72
	**Day 15**	95·0 (78·8-105·3; n=18)	91·0 (72·8-117·0; n=16)	0·85
FiO_2	**Baseline**	24·0 (21·0-32·0; n=88)	24·0 (21·0-30·8; n=80)	0·57
	**Day 15**	21·0 (21·0-32·0; n=36)	23·5 (21·0-50·0; n=30)	0·41
PaO_2/FiO_2	**Baseline**	276·0 (209·5-318·9; n=56)	278·6 (223·8-306·0; n=55)	0·88
	**Day 15**	440·5 (306·9-501·2; n=18)	270·3 (215·0-428·6; n=15)	0·06
SpO_2/FiO_2	**Baseline**	380·0 (280·0-440·0; n=86)	380·0 (300·0-440·0; n=75)	0·41
	**Day 15**	450·0 (310·0-470·0; n=33)	450·0 (210·0-460·0; n=27)	0·83

© 2023 Fanlo P et al. *JAMA Network Open.*

eTable 8. Levels of Hyperinflammatory Parameters and Other Relevant Laboratory Parameters by Timepoint

Variable	**Baseline** (randomisation)			**Day 4**			**Day 7**			**Day 15**		
	**Anakinra**	**Standard of care**	**p-value**	**Anakinra**	**Standard of care**	**p-value**	**Anakinra**	**Standard of care**	**p-value**	**Anakinra**	**Standard of care**	**p-value**
Hyperinflammatory parameters												
C-reactive protein	93·3 (36·4-155·7; n=88)	87·1 (39·2-146·6; n=86)	0·75	90·5 (34·1-155·7; n=88)	86·3 (39·7-149·4; n=85)	0·99	6·1 (2·5-21·1; n=77)	8·6 (3·9-23·7; n=61)	0·29	1·4 (0·6-7·4; n=51)	4·0 (1·3-18·1; n=51)	0·02
D-dimer	607·0 (428·5-984·0; n=81)	608·0 (375·0-1030·0; n=83)	0·95	699·0 (450·0-1290; n=83)	600·0 (358·3-1104·8; n=74)	0·13	829·0 (460·0-1327·0; n=75)	650·0 (362·0-1594·0; n=59)	0·26	468·0 (314·0-1027·5; n=49)	500·0 (300·0-955·0; n=51)	0·97
Ferritin	976·0 (452·5-1437·5;	803·5 (443·8-1365·2; n=84)	0·44	863·0 (346·0-	599·5 (393·8-1183·7; n=72)	0·22	755·2 (350·4-	670·5 (451·0-1068·9; n=55)	0·72	440·0 (208·0-749·0; n=49	392·5 (246·8-	0·57
IL-6	16·0 (5·2-47·2; n=61)	17·0 (6·7-46·9; n=53)	0·81	5·7 (3·4-21·3; n=56)	6·7 (3·1-22·8; n=57)	0·99	4·7 (2·8-11·3; n=46)	5·0 (2·4-24·9; n=43)	0·89	3·8 (2·7-8·9; n=32)	5·0 (2·7-9·5; n=33)	0·84
LDH	389·0 (310·0-500·3; n=86)	376·0 (291·5-486·0; n=85)	0·57	325·5 (261·3-448·5; n=80)	351·0 (234·0-413·0; n=77)	0·66	306·0 (248·5-427·0; n=77)	301·5 (238·8-410·8; n=58)	0·63	236·0 (202·3-299·8; n=52)	233·0 (199·0-276·3; n=46)	0·66
Other relevant laboratory parameters												
White blood cell count	6·7 (5·3-9·5; n=88)	7·7 (5·0-10·1; n=86)	0·31	6·7 (5·4-9·3; n=88)	7·3 (5·0-10·1; n=86)	0·42	9·3 (7·3-11·7; n=76)	9·5 (7·9-12·6; n=64)	0·18	7·3 (5·2-9·7; n=54)	8·6 (6·5-11·6; n=52)	0·07
Lymphocyte count	0·80 (0·56-1·20; n=88)	0·88 (0·63-1·21; n=86)	0·37	0·80 (0·55-1·28; n=88)	0·80 (0·62-1·20; n=86)	0·51	1·42 (0·77-2·17; n=76)	1·53 (0·97-2·29; n=64)	0·48	1·72 (0·97-2·26; n=54)	1·53 (1·12-2·38; n=52)	0·54
Platelet count	225·5 (177·8-302·5; n=88)	239·5 (183·8-325·5; n=86)	0·26	225·5 (177·8-302·0; n=88)	240·5 (183·8-325·5; n=86)	0·27	312·0 (250·5-439·8; n=76)	384·0 (305·0-472·0; n=63)	0·03	209·0 (170·5-293·5; n=54)	268·0-213·5-360·0; n=52)	0·01
Haemoglobin	14·1 (13·1-15·2; n=88)	13·8 (12·6-14·8; n=86)	0·26	14·1 (13·1-15·2; n=88)	13·8 (12·6-14·7; n=86)	0·19	13·8 (12·5-14·9; n=76)	13·8 (12·3-14·9; n=64)	0·82	13·1 (11·9-14·4; n=54)	13·1 (11·7-14·2; n=52)	0·89
RBC	4·7 (4·3-5·0; n=88)	4·7 (4·3-5·0; n=86)	0·33	4·7 (4·4-5·1; n=88)	4·6 (4·3-4·9; n=86)	0·15	4·6 (4·2-5·0; n=76)	4·6 (4·1-5·; n=64)	0·66	4·3 (3·9-4·8; n=76)	4·4 (3·9-4·7; n=52)	0·99

© 2023 Fanlo P et al. *JAMA Network Open.*

ESR	47·0 (30·0-63·0; n=21)	50·0 (29·0-88·0; n=21)	0·38	44·0 (30·8-66·3; n=26)	54·0 (29·0-88·0; n=25)	0·29	37·0 (18·0-55·0; n=27)	50·0 (27·0-77·8; n=16)	0·22	22·0 (7·0-46·0; n=23)	43·0 (10·3-51·8; n=16)	0·19
Fibrinogen	713·0 (621·5-785·8; n=76)	714·0 (609·5-819·8; n=80)	0·61	713·0 (604·5-	700·0 (599·3-804·0; n=80)	0·80	464·0 (401·5-	467·5 (406·5-602·5; n=58)	0·87	392·0 (303·8-482·8; n=48)	412·0 (348·0-	0·13
Prothrombin time	10·9 (1·1-12·6; n=82)	1·3 (1·1-12·6; n=84)	0·25	10·9 (1·1-13·0; n=82)	1·4 (1·1-12·8; n=84)	0·20	11·0 (1·0-12·3; n=76)	10·3 (1·0-12·3; n=61)	0·59	10·2 (1·1-11·3; n=52)	10·2 (1·0-11·4; n=51)	0·59
AST	36·5 (25·8-50·0; n=82)	39·0 (26·5-56·5; n=85)	0·25	36·0 (25·0-50·0; n=85)	38·0 (26·0-53·0; n=85)	0·35	32·0 (22·0-48·8; n=76)	30·0 (19·0-47·8; n=56)	0·84	27·0 (19·5-46·0; n=53)	27·0 (21·0-41·0; n=47)	0·82
ALT	35·0 (24·0-61·0; n=87)	38·5 (25·0-61·8; n=84)	0·59	34·0 (25·0-61·0; n=87)	40·0 (24·0-61·0; n=85)	0·74	51·5 (32·0-92·3; n=76)	65·0 (33·0-123·0; n=59)	0·15	56·0 (33·5-84·5; n=53)	60·5 (37·0-83·0; n=48)	0·66
Total bilirubin	0·5 (0·4-0·6; n=67)	0·5 (0·4-0·6; n=71)	0·45	0·5 (0·4-0·7; n=68)	0·5 (0·3-0·6; n=72)	0·12	0·5 (0·4-0·7; n=67)	0·5 (0·3-0·6; n=72)	0·91	0·6 (0·5-0·9; n=51)	0·6 (0·4-0·7; n=50)	0·06
Creatinine	0·8 (0·7-1·0; n=88)	0·8 (0·7-0·9; n=71)	0·08	0·8 (0·7-0·9; n=87)	0·8 (0·7-0·9; n=86)	0·17	0·8 (0·7-0·9; n=77)	0·8 (0·6-0·9; n=86)	0·87	0·8 (0·7-0·9; n=77)	0·7 (0·7-0·9; n=86)	0·57

Data are n (%) or median (IQR)

© 2023 Fanlo P et al. *JAMA Network Open.*

eTable 9. Evolution of Hyperinflammatory Parameters Over Time

Variable	Group	Baseline	Day 4	Day7	Day15	p-value Group	p-value Visit	p-value Group * visit
D-dimer	Anakinra	983·5 (1154·5; n=81)	1864·1 (4409·4; n=83)	2148·2 (5752·8; n=75)	1025·1 (1837·0; n=49)	0·82	0·40	0·66
	Standard of care	1618·7 (4356·3; n=83)	1953·6 (4730·0; n=74)	2048·6 (4469·9; n=59)	1846·3 (5114·3; n=51)			
Ferritin	Anakinra	1168·7 (969·7; n=85)	987·6 (789·3; n=83)	902·1 (768·5; n=75)	591·7 (512·2; n=49)	0·71	<0·001	0·64
	Standard of care	1153·4 (1168·4; n=84)	932·7 (1320·2; n=72)	902·8 (1303·6; n=55)	519·3 (409·7; n=46)			
Il-6	Anakinra	42·8 (73·4; n=61)	23·1 (41·3; n=56)	25·4 (73·3; n=46)	10·3 (15·9; n=32)	0·17	0·62	0·39
	Standard of care	49·4 (113·8; n=53)	52·2 (197·9; n=57)	42·2 (107·3; n=43)	65·1(287·1; n=33)			
LDH	Anakinra	416·5 (146·5; n=86)	355·5 (130·0; n=80)	352·8 (164·9, n=77)	263·2 (92·8; n=52)	0·94	<0·001	0·99
	Standard of care	415·9 (172·9; n=85)	357·1 (166·1; n=77)	335·5 (149·5, n=58)	265·3 (128·1; n=46)			
Fibrinogen	Anakinra	718·8 (187·7; n=76)	714·0 (176·2; n=78)	498·1 (146·8; n=58)	431·6 (172·2; n=48)	0·23	<0·001	0·98
	Standard of care	746·3 (200·2; n=80)	735·0 (197·1; n=80)	511·4 (166·5; n=58)	479·8 (182·0; n=47)			
CRP	Anakinra	104·2 (79·3; n=88)	102·3 (79·5; n=88)	25·4 (47·7; n=77)	13·3 (36·5; n=54)	0·96	<0·001	0·73
	Standard of care	102·2 (81·6; n=86)	103·5 (82·6; n=85)	25·3 (38·2; n=61)	22·3 (48·7; n=51)			
PaO2/FiO2	Anakinra	2·6 (0·9; n=56)	4·0 (1·6; n=18)			0·39	<0·001	0·21
	Standard of care	2·7 (0·8; n=55)	3·0 (1·3; n=15)					

Data are mean values (with SD and n). P-values obtained using mixed effect models.

© 2023 Fanlo P et al. *JAMA Network Open.*

eTable 10. Primary and Secondary Efficacy Outcomes in Patients With SCOPE Score ≥ 6

	Anakinra group (n=89)	Standard of care group (n=87)	RR or HR (95% CI)	p-value
**Primary endpoint**				
Patients not requiring mechanical ventilation up to day 15	41 (71·9%; n=57)	41 (82·0%; n=50)	RR 0·88 (0·71-1·08)	0·26*
Patients not requiring invasive mechanical ventilation up to day 15	47 (82·5%; n=57)	45 (84·9%; n=53)	RR 0·97 (0·82-1·15)	0·80
**Co-primary endpoints**				
Time to mechanical ventilation	NR (NR-NR; 59)	NR (NR-NR; 55)	HR 1·69 (0·74-3·81)	0·21†
Time to mechanical ventilation (death)	NR (NR-NR; 59)	NR (NR-NR; n=55)	RR 1 52 (0 69-3 34)	0·30
Oxygen supplementation until day 15‡	7 (100%; n=7)	9 (81·8%; n=11)	RR 1·22 (0·93-1·61)	0·50§
Hospital stay, days	10·0 (8·0-16·0; n=45)	9·0 (6·0-13·0; n=43)	..	0·0471¶
ICU admission	11 (19 3; n=57)	11 (20·8; n=53)	RR 0·93 (0·44-1·96)	1·00
ICU stay, days	12·5 (6 3·0-42·5; n=8)	20 (6·5-28·5; n=9)	..	0·85¶
**Secondary endpoints**				
Mortality at day 28	4 (7·0; n=57)	5 (9·4; n=53)	RR 0·74 (0·21-2·62)	0·74§
Mortality at 48 h since admission	0 (0·0)	0 (0·0)	..	..
Mortality at day 7 since admission	0 (0·0; n=4)	1 (1·7; n=5)	..	1·00§
Mortality at 48 h since ICU admission	0 (0·0)	0 (0·0)	..	..
Mortality at day 7 since ICU admission	1 (1·6; n=4)	0 (0·0; n=5)	..	0·44§
Negative PCR test	15 (45·5; n=33)	13 (43·3; n=30)	RR 1·05 (0·60–1·83)	1·00*

CI: confidence interval. ICU: Intensive Care Unit. PCR: polymerase chain reaction. RR: risk ratio. *Chi-square. †Log-rank test ‡Patients who were on supplemental oxygen at randomisation were excluded from the analyses (anakinra group: n=74, standard of care group: n=67), data were available for 15 patients in the anakinra group and 19 patients in the standard of care group. §Fisher [illegible]

© 2023 Fanlo P et al. *JAMA Network Open.*

eTable 11. Adjusted Multivariate Cox Regression Hazard Model to Estimate Risk Factors for Mechanical Ventilation

Variable	Adjusted model*	
	HR (95% CI)	p-value
Group of treatment (anakinra vs. standard of care)	1·84 (0·87-3·88)	0·11
COVID-19 severity (>5 vs. ≤5)	7·07 (3·22-15·55)	<0·001
Intake of dexamethasone (yes/no)	0·69 (0·30-1·62)	0·40

HR: hazard ratio. CI: confidence interval. *Cox regression model adjusted by treatment group, disease severity (WHO-CPS score), and intake of dexamethasone

© 2023 Fanlo P et al. *JAMA Network Open.*

eTable 12. Cox Regression Hazard Model to Estimate Risk Factors for Survival

	Unadjusted model		Adjusted model*	
**Variable**	**HR (95% CI)**	**p-value**	**HR (95% CI)**	**p-value**
Group of treatment (anakinra vs. standard of care)	0·78 (0·21-2·91)	0·714	1·02 (0·27-3·87)	0·98
COVID-19 severity (>5 vs. ≤5)	..	..	18·93 (3·39-105·58)	<0·001
Intake of dexamethasone (yes/no)	..	..	0·44 (0·09-2·00)	0·29

HR: hazard ratio. CI: confidence interval. *Cox regression model adjusted by treatment group, disease severity (WHO-CPS score), and intake of dexamethasone

© 2023 Fanlo P et al. *JAMA Network Open.*

eTable 13. Logistic Regression Analysis Model of Factors Affecting Viral Clearance

Variable	Univariate analysis		Multivariate adjusted model*	
	**OR (95% CI)**	**p-value**	**OR (95% CI)**	**p-value**
Group of treatment (anakinra vs. standard of care)	1·12 (0·51-2·47)	0·78	0·93 (0·41-2·09)	0·85
COVID-19 severity (>5 vs. ≤5)	1·24 (0·47-3·23)	0·66	2·37 (0·75-7·48)	0·14
Intake of dexamethasone (yes/no)	0·50 (0·22-1·11)	0·09	0·34 (0·13-0·90)	0·03

OR: odds ratio. CI: confidence interval. *Cox regression model adjusted by treatment group, disease severity (WHO-CPS score), and intake of dexamethasone

© 2023 Fanlo P et al. *JAMA Network Open.*

eTable 14. Rescue Therapy

	Anakinra group (n=89)	Standard of care group (n=87)
**Requirement of rescue therapy**	3(3·4; n=89)	16 (18·4; n=87)
**Rescue therapy**		
Tocilizumab	2 (66·7)	8 (50·1)
Hyperimmune plasma	1 (33·0)	3 (18·9)
Tocilizumab + hyperimmune plasma	0 (0·0)	1 (6·3)
Baricitinib	0 (0·0)	1 (6·3)

© 2023 Fanlo P et al. *JAMA Network Open.*

eTable 15. Imaging Assessment

	Baseline (screening)			Day 15		
	**Anakinra (n=88)**	**Standard of care (n=86)**	**p-value**	**Anakinra (n=53)**	**Standard of care (n=53)**	**p-value**
Imaging technique						
Chest X-ray	75 (85·2; n=88)	72 (83·7; n=86)	0·84*	45 (85·9; n=53)	51 (96·2; n=53)	0·09
HRCT	13 (14·8; n=88)	14 (16·3; n=86)		8 (15·1; n=53)	2 (3·8; n=53)	
Imaging assessment result						
Chest X-ray						
Normal	75 (100·0; n=75)	72 (100·0; n=72)	..	17 (37·8; n=45)	9 (17·6; n=51)	0·04
Abnormal	0 (0·0; n=75)	0 (0·0; n=72)	..	28 (62·2; n=45)	42 (82·4; n=51)	
HRCT						
Normal	13 (100·0; n=13)	14 (100·0; n=14)	..	1 (12·5; n=8)	0 (0·0; n=2)	..
Abnormal	0 (0·0; n=13)	0 (0·0; n=14)	..	7 (87·5; n=8)	2 (100·0; n=2)	
Extension						
Unilobar	10 (11·4; n=88)	10 (11·6; n=86)	1·00*	8 (22·9; n=35)	11 (25·0; n=44)	1·00*
Multilobar	78 (88·6; n=88)	76 (88·4; n=86)		27 (77·1; n=35)	33 (75·0; n=44)	
Lung sides						
Unilateral	8 (9·1; n=88)	4 (4·7; n=86)	0·37*	4 (11·4; n=35)	8 (18·2; n=44)	0·53*
Bilateral	80 (90·9; n=88)	82 (95·3; n=86)		31 (88·6; n=35)	36 (81·8; n=44)	
Diffuse	47 (53·4; n=88)	49 (57·0; n=86)	0·65*	13 (37·1; n=35)	18 (40·9; n=44)	0·82*
Interstitial infiltrates	67 (76·1; n=88)	56 (65·1; n=86)	0·13*	24 (68·6; n=35)	33 (75·0; n=44)	0·62*
Opacities	48 (54·5; n=88)	51 (59·3; n=86)	0·54*	15 (42·9; n=35)	23 (52·3; n=44)	0·50*
Pneumothorax	0 (0·0; n=88)	0 (0·0; n=86)	..	0 (0·0; n=35)	0 (0·0; n=44)	..
Pleural effusion	1 (1·1; n=88)	2 (2·3; n=86)	0·62†	1 (2·9; n=35)	1 (2·3; n=44)	1.00†
Ground grass infiltrates	12 (15·2; n=79)	21 (26·3; n=80)	0·12*	9 (27·3; n=33)	8 (18·6; n=43)	0·41*

Data are n (%). *Chi-square; †Fisher exact test. HRCT: High-resolution computed tomography

© 2023 Fanlo P et al. *JAMA Network Open.*

eTable 16. Symptoms of COVID-19 at Day 28

Symptoms	Anakinra group (n=52)	Standard of care group (n=53)
Presence of symptoms	40 (76·9; n=52)	40 (76·9; n=52)
Symptoms*		
Dyspnoea	24 (27·0)	24 (27·6)
Fatigue	18 (20·2)	19 (21·8)
Cough	9 (10·1)	6 (6·9)
Expectoration	6 (6·7)	4 (4·6)
Loss of appetite	4 (4·5)	4 (4·6)
Fever or low-grade fever	2 (2·2)	5 (5·7)
Chest pain	2 (2·2)	3 (3·4)
Myalgia	1 (1·1)	4 (4·6)
Dysgeusia	3 (3·4)	2 (2·3)
Arthralgia	2 (2·2)	2 (2·3)

Data are n (%). *Most common symptoms presented by at least 5% of patients each

© 2023 Fanlo P et al. *JAMA Network Open.*

eTable 17. Serious Adverse Events

Serious adverse events *	**Anakinra group (n=89)**	**Standard of care group (n=87)**
Acute coronary syndrome	1	0
Acute kidney injury	1	0
Acute pulmonary edema	1	0
Deep vein thrombosis	1	0
Endotracheal intubation	0	1
Hypoxia	0	1
Oxygen saturation decrease	1	0
Pneumonia	1	0
Pneumothorax	1	0
Psychotic disorder	1	0
Respiratory insufficiency	1	1
Respiratory tract infection bacterial	1	0
Superinfection bacterial	1	0
Respiratory distress	1	0
Renal failure	1	0
Urinary tract infection	0	1

* Serious adverse events reported in less than 2 patients each

© 2023 Fanlo P et al. *JAMA Network Open.*

eFigure 1. Evolution of the First Panel of Exploratory Outcomes

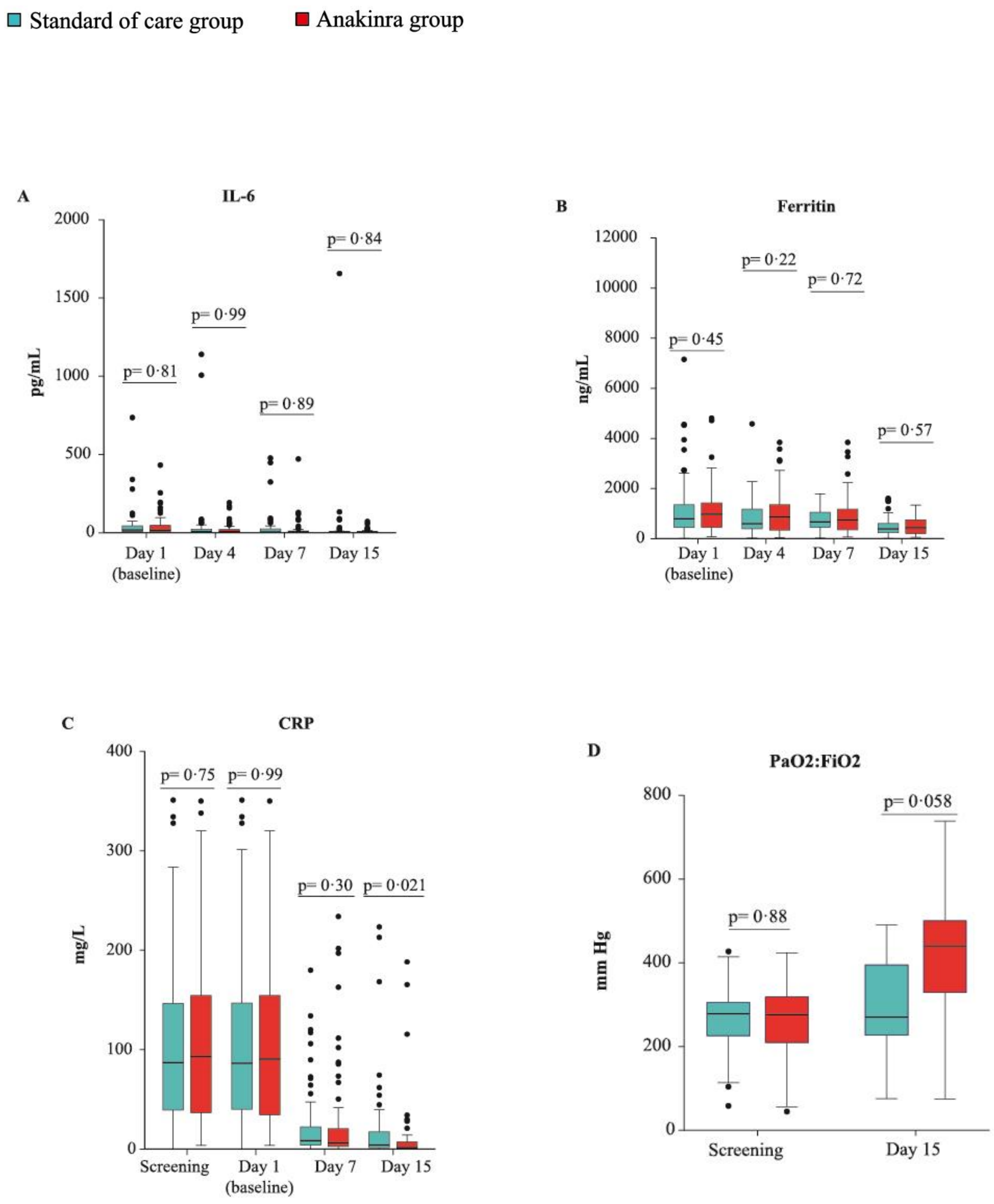

© 2023 Fanlo P et al. *JAMA Network Open.*

eFigure 2. Evolution of the Second Panel of Exploratory Outcomes

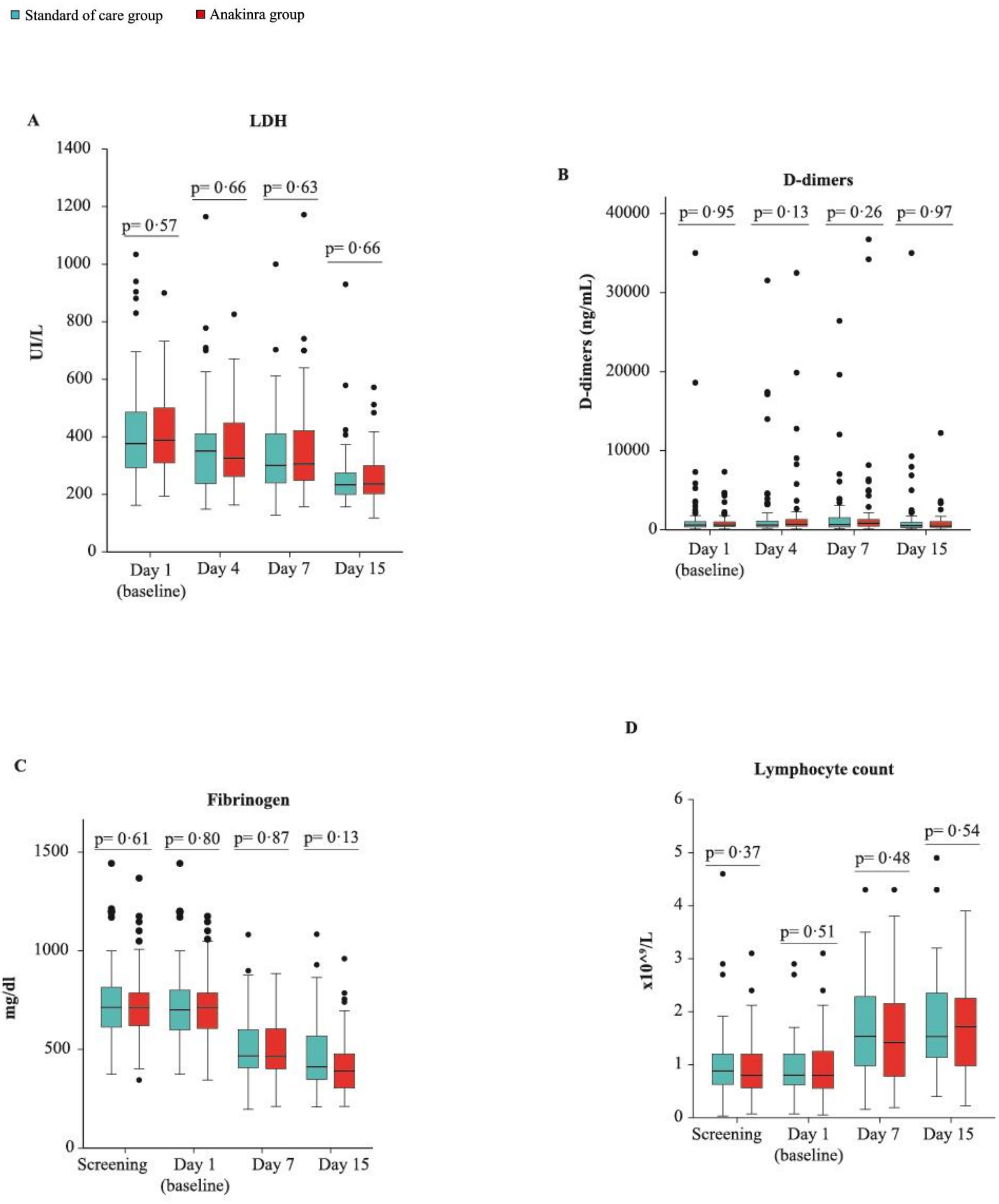

© 2023 Fanlo P et al. *JAMA Network Open.*

eReferences

1. Granowitz E et al. Pharmacokinetics, safety, and immunomodulatory effects of human recombinant interleukin-1 receptor antagonist in healthy humans. CYTOKINE 1992; 4(5):353-360
2. Badheka A et al. Use of an interleukin-1 receptor antagonist for suspected sepsis with hyperinflammation in children. Crit Care Med. 2019; 48:1561
3. Opal S et al. Confirmatory interleukin-1 receptor antagonist trial in severe sepsis: A phase III, randomized, double-blind, placebo-controlled, multicenter study. Crit Care Med. 1997; 25:1115-1124
4. Fisher C et al. Initial evalutation of human recombinant interleukin-1 receptor antagonist in the treatment of sepsis syndrome: A randomized, open-label, placebo-controlled multicenter study. CRITICAL CARE MEDICINE 1994; 22(1):12-21
5. Galea J et al. Intravenous anakinra can achieve experimentally effective concentrations in the central nervous system within a therapeutic time window results of a dose-ranging study. JCBFM.2011; 31:439-447

© 2023 Fanlo P et al. *JAMA Network Open.*
