# Supplementary material for: Efficacy and Safety of Anakinra Plus Standard of Care for Patients With Severe COVID-19: A Randomized Phase 2/3 Clinical Trial
Source: JAMA Netw Open. 2023 Apr 7;6(4):e237243. doi: 10.1001/jamanetworkopen.2023.7243 (PMC10082404; doi:10.1001/jamanetworkopen.2023.7243)
Supplement: Supplement 3. — GEAS-SEMI Nonauthor Collaborators [file jamanetwopen-e237243-s003.pdf]

*First name, last name, and suffix (if applicable) are required and will appear in PubMed.

***Group Name(s): GEAS-SEMI Group**

***First Name and Middle Initial(s)**	***Last Name**	***Suffix (eg, Jr, III)**	Academic Degrees	Institution	Location (city, state/province, country)	Role or Contribution, eg, chair, principal investigator	Group (if more than 1 Group listed in the byline) and/or Subgroup (eg, Steering Committee)
Rubén	Arnáez		MD	Unidad de Enfermedades Autoinmunes Sistémicas. Servicio de Medicina Interna. Hospital Universitario de Navarra. Navarrabiomed-HUN-UPNA.	Pamplona, Navarra (Spain)	Collaborator	GEAS-SEMI Group
Elisa	Huarte		MD	Unidad de Enfermedades Autoinmunes Sistémicas. Servicio de Medicina Interna. Hospital Universitario de Navarra. Navarrabiomed-HUN-UPNA.	Pamplona, Navarra (Spain)	Collaborator	GEAS-SEMI
Julio	Sanchez		MD, PhD	Unidad de Enfermedades Autoinmunes Sistémicas. Servicio de Medicina Interna. Hospital Universitario de Navarra. Navarrabiomed-HUN-UPNA.	Pamplona, Navarra (Spain)	Collaborator	GEAS-SEMI
Eva	Zabalza		MSc	Navarrabiomed-HUN-UPNA.	Pamplona, Navarra (Spain)	Collaborator	GEAS-SEMI
Ruth	Garcia-Rey		MsC	Navarrabiomed-HUN-UPNA.	Pamplona, Navarra (Spain)	Collaborator	GEAS-SEMI
Maria	Gonzalo		MD	Unidad de Enfermedades Autoinmunes Sistémicas. Servicio de Medicina Interna. Hospital Universitario de Navarra. Navarrabiomed-HUN-UPNA.	Pamplona, Navarra (Spain)	Collaborator	GEAS-SEMI
Laura	Diez-Galán		MD	Unidad de Enfermedades Autoinmunes Sistémicas. Servicio de Medicina Interna. Hospital Universitario Clínico Lozano Blesa.Instituto de Investigación Sanitaria de Aragón	Zaragoza, Aragón (Spain)	Collaborator	GEAS-SEMI

Supplemental Online Content: Nonauthor Collaborators
*First name, last name, and suffix (if applicable) are required and will appear in PubMed.

***First Name and Middle Initial(s)**	***Last Name**	***Suffix (eg, Jr, III)**	Academic Degrees	Institution	Location (city, state/province, country)	Role or Contribution, eg, chair, principal investigator	Group (if more than 1 Group listed in the byline) and/or Subgroup (eg, Steering Committee)
Marisa	de la Rica-Escuín		MD	Unidad de Enfermedades Autoinmunes Sistémicas. Servicio de Medicina Interna. Hospital Universitario Clínico Lozano Blesa.Instituto de Investigación Sanitaria de Aragón	Zaragoza, Aragón (Spain)	Collaborator	GEAS-SEMI
Luis	Martinez-Lostao		MD	Unidad de Enfermedades Autoinmunes Sistémicas. Servicio de Medicina Interna. Hospital Universitario Clínico Lozano Blesa.Instituto de Investigación Sanitaria de Aragón	Zaragoza, Aragón (Spain)	Collaborator	GEAS-SEMI
Adela	Marín Ballvé		MD	Unidad de Enfermedades Autoinmunes Sistémicas. Servicio de Medicina Interna. Hospital Universitario Clínico Lozano Blesa.Instituto de Investigación Sanitaria de Aragón	Zaragoza,Aragón (Spain)	Collaborator	GEAS-SEMI
María Luisa	Taboada-Martínez		MD	Unidad de Enfermedades Autoinmunes Sistémicas. Servicio de Medicina Interna. Hospital de Cabueñes.	Gijón,Asturias (Spain)	Collaborator	GEAS-SEMI
Rubén	Pampín-Sánchez		MD	Unidad de Enfermedades Autoinmunes Sistémicas. Servicio de Medicina Interna. Hospital de Cabueñes.	Gijón,Asturias (Spain)	Collaborator	GEAS-SEMI
Cristina	Helguera-Amézua		MD	Unidad de Enfermedades Autoinmunes Sistémicas. Servicio de Medicina Interna. Hospital de Cabueñes.	Gijón,Asturias (Spain)	Collaborator	GEAS-SEMI

Supplemental Online Content: Nonauthor Collaborators
*First name, last name, and suffix (if applicable) are required and will appear in PubMed.

***First Name and Middle Initial(s)**	***Last Name**	***Suffix (eg, Jr, III)**	Academic Degrees	Institution	Location (city, state/province, country)	Role or Contribution, eg, chair, principal investigator	Group (if more than 1 Group listed in the byline) and/or Subgroup (eg, Steering Committee)
Rosa	Fernández-Madera-Martínez		MD	Unidad de Enfermedades Autoinmunes Sistémicas. Servicio de Medicina Interna. Hospital de Cabueñes.	Gijón,Asturias (Spain)	Collaborator	GEAS-SEMI
Estela	García-Coya		MD	Unidad de Enfermedades Autoinmunes Sistémicas. Servicio de Medicina Interna. Hospital de Cabueñes.	Gijón,Asturias (Spain)	Collaborator	GEAS-SEMI
Ana María	Álvarez-Suarez		MD	Unidad de Enfermedades Autoinmunes Sistémicas. Servicio de Medicina Interna. Hospital de Cabueñes.	Gijón,Asturias (Spain)	Collaborator	GEAS-SEMI
Ángel	Robles		MD	Unidad de Enfermedades Autoinmunes Sistémicas. Servicio de Medicina Interna. Hospital La Paz.	Madrid (Spain)	Collaborator	GEAS-SEMI
Ana	Noblejas		MD	Unidad de Enfermedades Autoinmunes Sistémicas. Servicio de Medicina Interna. Hospital La Paz.	Madrid (Spain)	Collaborator	GEAS-SEMI
Clara	Soto		MD	Unidad de Enfermedades Autoinmunes Sistémicas. Servicio de Medicina Interna. Hospital La Paz.	Madrid (Spain)	Collaborator	GEAS-SEMI
Elena	Martínez		MD	Unidad de Enfermedades Autoinmunes Sistémicas. Servicio de Medicina Interna. Hospital La Paz.	Madrid (Spain)	Collaborator	GEAS-SEMI
Francisco	Arnalich		MD	Unidad de Enfermedades Autoinmunes Sistémicas. Servicio de Medicina Interna. Hospital La Paz.	Madrid (Spain)	Collaborator	GEAS-SEMI

*First name, last name, and suffix (if applicable) are required and will appear in PubMed.

***First Name and Middle Initial(s)**	***Last Name**	***Suffix (eg, Jr, III)**	Academic Degrees	Institution	Location (city, state/province, country)	Role or Contribution, eg, chair, principal investigator	Group (if more than 1 Group listed in the byline) and/or Subgroup (eg, Steering Committee)
Coral	Arévalo		MD	Unidad de Enfermedades Autoinmunes Sistémicas. Servicio de Medicina Interna. Hospital Ramón y Cajal.	Madrid (Spain)	Collaborator	GEAS-SEMI
Angélica	López-Rodríguez		MD	Unidad de Enfermedades Autoinmunes Sistémicas. Servicio de Medicina Interna. Hospital Ramón y Cajal.	Madrid (Spain)	Collaborator	GEAS-SEMI
Pilar	Cobeta		MD	Unidad de Enfermedades Autoinmunes Sistémicas. Servicio de Medicina Interna. Hospital Ramón y Cajal.	Madrid (Spain)	Collaborator	GEAS-SEMI
Fernando	Hidalgo		MD	Unidad de Enfermedades Autoinmunes Sistémicas. Servicio de Medicina Interna. Hospital Ramón y Cajal.	Madrid (Spain)	Collaborator	GEAS-SEMI
Sergio	Diz		MD	Unidad de Enfermedades Autoinmunes Sistémicas. Servicio de Medicina Interna. Hospital Ramón y Cajal.	Madrid (Spain)	Collaborator	GEAS-SEMI
Paula	González		MD	Unidad de Enfermedades Autoinmunes Sistémicas. Servicio de Medicina Interna. Hospital Ramón y Cajal.	Madrid (Spain)	Collaborator	GEAS-SEMI
Nuria	Bara		MD	Unidad de Enfermedades Autoinmunes Sistémicas. Servicio de Medicina Interna. Hospital Ramón y Cajal.	Madrid (Spain)	Collaborator	GEAS-SEMI
Martin	Fabregate		MD	Unidad de Enfermedades Autoinmunes Sistémicas. Servicio de Medicina Interna. Hospital Ramón y Cajal.	Madrid (Spain)	Collaborator	GEAS-SEMI

Supplemental Online Content: Nonauthor Collaborators
*First name, last name, and suffix (if applicable) are required and will appear in PubMed.

*First Name and Middle Initial(s)	*Last Name	*Suffix (eg, Jr, III)	Academic Degrees	Institution	Location (city, state/province, country)	Role or Contribution, eg, chair, principal investigator	Group (if more than 1 Group listed in the byline) and/or Subgroup (eg, Steering Committee)
Judith	Jiménez		MD	Unidad de Enfermedades Autoinmunes Sistémicas. Servicio de Medicina Interna. Hospital Ramón y Cajal.	Madrid (Spain)	Collaborator	GEAS-SEMI
Svetlana	Zhilina		MD	Unidad de Enfermedades Autoinmunes Sistémicas. Servicio de Medicina Interna. Hospital Ramón y Cajal.	Madrid (Spain)	Collaborator	GEAS-SEMI
Martina	Pellicer-Ariño		MD	Servicio de Medicina Interna. Hospital Clinic.	Barcelona,Cataluña (Spain)	Collaborator	GEAS-SEMI
Olga	Rodríguez - Núñez		MD	Servicio de Medicina Interna. Hospital Clinic.	Barcelona,Cataluña (Spain)	Collaborator	GEAS-SEMI
Joan	Ribot - Grabalosa		MD	Servicio de Medicina Interna. Hospital Clinic.	Barcelona,Cataluña (Spain)	Collaborator	GEAS-SEMI
Míriam	Costafreda-Mas		MD	Servicio de Medicina Interna. Hospital Clinic.	Barcelona,Cataluña (Spain)	Collaborator	GEAS-SEMI
Adrián	Tomé-Pérez		MD	Servicio de Medicina Interna. Hospital Clinic.	Barcelona,Cataluña (Spain)	Collaborator	GEAS-SEMI
Teresa	Hospital-Vidal		MD	Servicio de Medicina Interna. Hospital Clinic.	Barcelona,Cataluña (Spain)	Collaborator	GEAS-SEMI
Andrea	Ladino-Vázquez		MD	Servicio de Medicina Interna. Hospital Clinic.	Barcelona,Cataluña (Spain)	Collaborator	GEAS-SEMI
Alma	Morancho-Sesé		MD	Servicio de Medicina Interna. Hospital Clinic.	Barcelona,Cataluña (Spain)	Collaborator	GEAS-SEMI
Adelaido	Salazar-Rustarazo		MD	Servicio de Medicina Interna. Hospital Clinic.	Barcelona,Cataluña (Spain)	Collaborator	GEAS-SEMI
Cristina	Gabara-Xancó		MD	Servicio de Medicina Interna. Hospital Clinic.	Barcelona,Cataluña (Spain)	Collaborator	GEAS-SEMI
Arturo	Gonzalez - Quintela		MD	Unidad de Enfermedades Autoinmunes Sistémicas. Servicio de Medicina Interna. Complejo Hospitalario de Santiago	Santiago de Compostela, Galicia (Spain)	Collaborator	GEAS-SEMI

Supplemental Online Content: Nonauthor Collaborators
*First name, last name, and suffix (if applicable) are required and will appear in PubMed.

***First Name and Middle Initial(s)**	***Last Name**	***Suffix (eg, Jr, III)**	Academic Degrees	Institution	Location (city, state/province, country)	Role or Contribution, eg, chair, principal investigator	Group (if more than 1 Group listed in the byline) and/or Subgroup (eg, Steering Committee)
Bernardo	Sopeña		MD	Unidad de Enfermedades Autoinmunes Sistémicas. Servicio de Medicina Interna. Complejo Hospitalario de Santiago	Santiago de Compostela, Galicia (Spain)	Collaborator	GEAS-SEMI
Rosario	Alende -Sixto		MD	Unidad de Enfermedades Autoinmunes Sistémicas. Servicio de Medicina Interna. Complejo Hospitalario de Santiago	Santiago de Compostela, Galicia (Spain)	Collaborator	GEAS-SEMI
Helena	Esteban		MD	Unidad de Enfermedades Autoinmunes Sistémicas. Servicio de Medicina Interna. Complejo Hospitalario de Santiago	Santiago de Compostela, Galicia (Spain)	Collaborator	GEAS-SEMI
Nuria	Rodriguez-Nuñez		MD	Unidad de Enfermedades Autoinmunes Sistémicas. Servicio de Medicina Interna. Complejo Hospitalario de Santiago	Santiago de Compostela, Galicia (Spain)	Collaborator	GEAS-SEMI
Ariadna Helena	Andrade-Piña		MD	Unidad de Enfermedades Autoinmunes Sistémicas. Servicio de Medicina Interna. Complejo Hospitalario de Santiago	Santiago de Compostela, Galicia (Spain)	Collaborator	GEAS-SEMI
Maria Ángeles	Sanchidrian-Chapinal		MD	Unidad de Enfermedades Autoinmunes Sistémicas. Servicio de Medicina Interna. Complejo Hospitalario de Santiago	Santiago de Compostela, Galicia (Spain)	Collaborator	GEAS-SEMI
Pablo	Varela		MD	Unidad de Enfermedades Autoinmunes Sistémicas. Servicio de Medicina Interna. Complejo Hospitalario de Santiago	Santiago de Compostela, Galicia (Spain)	Collaborator	GEAS-SEMI
Manuel	Taboada		MD	Unidad de Enfermedades Autoinmunes Sistémicas. Servicio de Medicina Interna. Complejo Hospitalario de Santiago	Santiago de Compostela, Galicia (Spain)	Collaborator	GEAS-SEMI

Supplemental Online Content: Nonauthor Collaborators
*First name, last name, and suffix (if applicable) are required and will appear in PubMed.

***First Name and Middle Initial(s)**	***Last Name**	***Suffix (eg, Jr, III)**	Academic Degrees	Institution	Location (city, state/province, country)	Role or Contribution, eg, chair, principal investigator	Group (if more than 1 Group listed in the byline) and/or Subgroup (eg, Steering Committee)
Brenda	Maure-Noia		MD	Unidad de Enfermedades Autoinmunes Sistémicas. Servicio de Medicina Interna. Complejo Hospitalario de Vigo	Vigo, Galicia (Spain)	Collaborator	GEAS-SEMI
Ana	López-Domínguez		MD	Unidad de Enfermedades Autoinmunes Sistémicas. Servicio de Medicina Interna. Complejo Hospitalario de Vigo	Vigo, Galicia (Spain)	Collaborator	GEAS-SEMI
Carmen	Filloy-Mato		MD	Unidad de Enfermedades Autoinmunes Sistémicas. Servicio de Medicina Interna. Complejo Hospitalario de Vigo	Vigo, Galicia (Spain)	Collaborator	GEAS-SEMI
Beatriz	Gimena-Reyes		MD	Unidad de Enfermedades Autoinmunes Sistémicas. Servicio de Medicina Interna. Complejo Hospitalario de Vigo	Vigo, Galicia (Spain)	Collaborator	GEAS-SEMI
Marisol	Samartín-Ucha		MD	Unidad de Enfermedades Autoinmunes Sistémicas. Servicio de Medicina Interna. Complejo Hospitalario de Vigo	Vigo, Galicia (Spain)	Collaborator	GEAS-SEMI
Caritina	Vázquez-Triñanes		MD	Unidad de Enfermedades Autoinmunes Sistémicas. Servicio de Medicina Interna. Complejo Hospitalario de Vigo	Vigo, Galicia (Spain)	Collaborator	GEAS-SEMI
Julian	Fernández-Martín		MD	Unidad de Enfermedades Autoinmunes Sistémicas. Servicio de Medicina Interna. Complejo Hospitalario de Vigo	Vigo, Galicia (Spain)	Collaborator	GEAS-SEMI
Adolfo	Paradela-Carreiro		MD	Unidad de Enfermedades Autoinmunes Sistémicas. Servicio de Medicina Interna. Complejo Hospitalario de Vigo	Vigo, Galicia (Spain)	Collaborator	GEAS-SEMI

*First name, last name, and suffix (if applicable) are required and will appear in PubMed.

***First Name and Middle Initial(s)**	***Last Name**	***Suffix (eg, Jr, III)**	Academic Degrees	Institution	Location (city, state/province, country)	Role or Contribution, eg, chair, principal investigator	Group (if more than 1 Group listed in the byline) and/or Subgroup (eg, Steering Committee)
Ana María	Regueira-Arcay		MD	Unidad de Enfermedades Autoinmunes Sistémicas. Servicio de Medicina Interna. Complejo Hospitalario de Vigo	Vigo, Galicia (Spain)	Collaborator	GEAS-SEMI
Eva	Esteban-Marcos		MD	Unidad de Enfermedades Autoinmunes Sistémicas. Servicio de Medicina Interna. Hospital Son Espases.	Palma de Mallorca.Islas Bareales (Spain)	Collaborator	GEAS-SEMI
Luisa	Martin-Pena		MD	Unidad de Enfermedades Autoinmunes Sistémicas. Servicio de Medicina Interna. Hospital Son Espases.	Palma de Mallorca, Islas Baleares (Spain)	Collaborator	GEAS-SEMI
Juan Antonio	Fernández-Navarro		MD	Unidad de Enfermedades Autoinmunes Sistémicas. Servicio de Medicina Interna. Hospital Universitario La Fe.	Valencia (Spain)	Collaborator	GEAS-SEMI
Noelia	Abdilla-Bonias		MD	Unidad de Enfermedades Autoinmunes Sistémicas. Servicio de Medicina Interna. Hospital Universitario La Fe.	Valencia (Spain)	Collaborator	GEAS-SEMI
Jaume	Mestre-Torres		MD	Unidad de Enfermedades Autoinmunes Sistémicas. Servicio de Medicina Interna. Hospital Vall d´Hebron.	Barcelona,Cataluña (Spain)	Collaborator	GEAS-SEMI
Joana Rita	Marques-Soares		MD	Unidad de Enfermedades Autoinmunes Sistémicas. Servicio de Medicina Interna. Hospital Vall d´Hebron.	Barcelona,Cataluña (Spain)	Collaborator	GEAS-SEMI
Josep	Pardos-Gea		MD	Unidad de Enfermedades Autoinmunes Sistémicas. Servicio de Medicina Interna. Hospital Vall d´Hebron.	Barcelona,Cataluña (Spain)	Collaborator	GEAS-SEMI
