# Supplementary material for: Efficacy and Safety of Anakinra Plus Standard of Care for Patients With Severe COVID-19: A Randomized Phase 2/3 Clinical Trial
Source: JAMA Netw Open. 2023 Apr 7;6(4):e237243. doi: 10.1001/jamanetworkopen.2023.7243 (PMC10082404; doi:10.1001/jamanetworkopen.2023.7243)
Supplement: Supplement 4. — Data Sharing Statement [file jamanetwopen-e237243-s004.pdf]

Data Sharing Statement

Fanlo. Efficacy and Safety of Anakinra Plus Standard of Care for Patients With Severe COVID-19. *JAMA Netw Open*. Published April 07, 2023. doi:10.1001/jamanetworkopen.2023.7243

Data
Data available: Yes
Data types: Participant data with identifiers
How to access data: patriciafanlo@yahoo.es
When available: With publication

Supporting Documents
Document types: Statistical/analytic code, Informed consent form
How to access documents: patriciafanlo@yahoo.es
When available: With publication

Additional Information
Who can access the data: Anyone requesting the data
Types of analyses: For any purpose
Mechanisms of data availability: With a signed data access agreement
